# A paneukaryotic genomic analysis of the small GTPase RABL2 underscores the significance of recurrent gene loss in eukaryote evolution

**DOI:** 10.1186/s13062-016-0107-8

**Published:** 2016-02-02

**Authors:** Marek Eliáš, Vladimír Klimeš, Romain Derelle, Romana Petrželková, Jan Tachezy

**Affiliations:** Department of Biology and Ecology, University of Ostrava, Faculty of Science, Chittussiho 10, 710 00 Ostrava, Czech Republic; Unité d’Ecologie, Systématique et Evolution, Centre National de la Recherche Scientifique UMR 8079, Université Paris-Sud, 91405 Orsay, France; Department of Parasitology, Charles University in Prague, Faculty of Science, Viničná 7, 128 44 Prague 2, Czech Republic

**Keywords:** Cilium, Evolution, Flagellum, Gene loss, Last eukaryotic common ancestor, Protists, Rab-like GTPases, RABL2

## Abstract

**Background:**

The cilium (flagellum) is a complex cellular structure inherited from the last eukaryotic common ancestor (LECA). A large number of ciliary proteins have been characterized in a few model organisms, but their evolutionary history often remains unexplored. One such protein is the small GTPase RABL2, recently implicated in the assembly of the sperm tail in mammals.

**Results:**

Using the wealth of currently available genome and transcriptome sequences, including data from our on-going sequencing projects, we systematically analyzed the phylogenetic distribution and evolutionary history of RABL2 orthologs. Our dense taxonomic sampling revealed the presence of RABL2 genes in nearly all major eukaryotic lineages, including small “obscure” taxa such as breviates, ancyromonads, malawimonads, jakobids, picozoans, or palpitomonads. The phyletic pattern of RABL2 genes indicates that it was present already in the LECA. However, some organisms lack RABL2 as a result of secondary loss and our present sampling predicts well over 30 such independent events during the eukaryote evolution. The distribution of RABL2 genes correlates with the presence/absence of cilia: not a single well-established cilium-lacking species has retained a RABL2 ortholog. However, several ciliated taxa, most notably nematodes, some arthropods and platyhelminths, diplomonads, and ciliated subgroups of apicomplexans and embryophytes, lack RABL2 as well, suggesting some simplification in their cilium-associated functions. On the other hand, several algae currently unknown to form cilia, e.g., the “prasinophytes” of the genus *Prasinoderma* or the ochrophytes *Pelagococcus subviridis* and *Pinguiococcus pyrenoidosus*, turned out to encode not only RABL2, but also homologs of some hallmark ciliary proteins, suggesting the existence of a cryptic flagellated stage in their life cycles. We additionally obtained insights into the evolution of the RABL2 gene architecture, which seems to have ancestrally consisted of eight exons subsequently modified not only by lineage-specific intron loss and gain, but also by recurrent loss of the terminal exon encoding a poorly conserved C-terminal extension.

**Conclusions:**

Our comparative analysis supports the notion that RABL2 is an ancestral component of the eukaryotic cilium and underscores the still underappreciated magnitude of recurrent gene loss, or reductive evolution in general, in the history of eukaryotic genomes and cells.

**Reviewers:**

This article was reviewed by Berend Snel and James O. McInerney.

**Electronic supplementary material:**

The online version of this article (doi:10.1186/s13062-016-0107-8) contains supplementary material, which is available to authorized users.

## Background

Cilia, flagella or undulipodia are different terms applied to the same basic cellular structure of the eukaryotic cell characterized, in its typical form, as a slender, plasma membrane-covered cell projection based on the axoneme – an actively bending bundle of microtubules emanating from the basal body and arranged in the characteristic 9 × 2 + 2 configuration [1]. Although the structural and functional complexity of cilia (for simplicity hereafter used as a synonym for flagella) had been appreciated for a very long time, the molecular underpinnings of the cilium biogenesis and functioning remained poorly understood until quite recently. Only in the past fifteen years or so, our knowledge on the protein composition of different ciliary substructures and molecular mechanisms involved in the assembly and maintenance of the cilium has grown significantly, primarily thanks to studies of mutants with cilia-associated phenotypes, proteomic investigations of isolated cilia and their substructures, and detailed biochemical and cell biological studies of individual ciliary proteins (reviewed, e.g., in [2, 3]). An important motivation behind the research on cilia has been the realization that perturbed structure or function of cilia is a cause of many human congenital diseases collectively called ciliopathies [4].

Significantly, the progress in identifying ciliary proteins has relied not only on experimental approaches, but has been strongly aided by bioinformatic analyses of genome sequences in the frame of comparative genomics. Nature affords us to use such a methodology owing to the fact that a number of eukaryotic lineages have independently lost the ability to build cilia, which in a typical case is accompanied by the loss of genes with cilium-specific functions (hereafter called ciliary genes). Looking for genes shared by ciliated organisms yet lacking in those devoid of cilia thus has a potential to uncover unknown ciliary genes. Indeed, the power of this approach has been demonstrated by numerous studies validating a ciliary role for candidate genes identified by comparative analyses (e.g., [5–7]).

Among the many structural classes of ciliary proteins one of the most prominent is the Ras superfamily of GTPases, often also called small GTPases [3, 8–10]. While some small GTPases functionally connected to the cilium have also other roles in the cell, and hence are not restricted to ciliated species, e.g., RAB8 or RAN [11], a growing list of GTPases seems to be specific for the cilium. The latter category includes ARL6/BBS3 [6, 12], IFT27/RABL4/RAYL [13, 14], IFT22/RABL5/IFTA-2/FAP9 [15–17], RAB23 [18, 19], ARL13B/ARL-13 [20, 21], ARL3 [21, 22], and RSG1 [23, 24]. Phylogenetic surveys were performed for some of these GTPases, and although limited in their scope, they suggested that these proteins are restricted to ciliated species. Based on a similar phyletic pattern, a cilium-related function was proposed also for small GTPases of the RJL family [25]. The RJL protein in *Trypanosoma cruzi* seems to localize to the flagellar pocket [26], which would be consistent with the aforementioned prediction. However, a recent investigation of the human member of the family, RBJ (or DNAJC27 according to the official human gene nomenclature), showed that it is a nuclear protein interacting with protein kinases and has a possible role in tumor progression [27]. Hence, the status of RJL/RBJ as ciliary GTPases remains uncertain.

The list of cilium-associated small GTPases was recently expanded by adding RABL2. Two virtually identical paralogs of this gene, RABL2A and RABL2B, were described a long time ago [28], but their cellular function had remained elusive until Lo et al. demonstrated that the single mouse ortholog, RABL2, is essential for sperm tail assembly and function [29]. The RABL2 protein localized to the sperm tail and interacted with components of the intraflagellar transport (IFT) complex B. Furthermore, several putative effectors preferentially binding the GTP-bound form of the protein were identified, and investigation of developing sperm from a mouse mutant exhibiting a defective version of the RABL2 protein suggested that RABL2 mediates delivery of these effector proteins to the growing tail. Together with the fact that expression of the RABL2 gene in mouse was biased towards tissues containing motile cilia, the authors suggested that the human RABL2 gene may be involved in a group of diseases called primary ciliary dyskinesia [29]. Indeed, mutation in the human RABL2A gene has been recently identified as a risk factor for oligoasthenospermic infertility in men [30].

Hints for a possible functional association of RABL2 homologs with cilia were actually available even before the study by Lo et al. [29]. Specifically, the RABL2 ortholog of *Chlamydomonas reinhardtii* was identified as a potential component of the flagella of this alga, based on its detection by a single peptide (see Table S2 in [31]), and the RABL2 protein was found in the proteome of the mouse photoreceptor sensory cilium complex [32]. Transcription of both RABL2A and RABL2B genes was up-regulated in human bronchial epithelial cells during mucociliary differentiation, along with many genes known to be involved in cilia formation [33]. Significantly for the present paper, RABL2 was included in the CiliaCut, a list of 186 protein families defined by a comparative genomic screen looking for genes shared by four ciliated species (the green alga *C. reinhardtii*, humans, and two *Phytophthora* species), but absent from a selected set of cilium-lacking species (see table SB in [34]). Some of these observations made a basis for listing the human RABL2A and RABL2B as potential ciliary genes in the SYSCILIA gold standard version 1 (SCGSv1) database [35].

While analyzing sequence data from our on-going genome and transcriptome sequencing projects for several interesting eukaryotic species – the anaerobic amoebozoan *Mastigamoeba balamuthi*, the eustigmatophyte alga *Trachydiscus minutus*, the jakobid *Andalucia godoyi*, and the malawimonads *Malawimonas californiana* and *Malawimonas* sp. strain 249, we noticed the presence of candidate RABL2 orthologs in these organisms. These were significant observations: (1) the presence of RABL2 in *M. balamuthi* was exceptional among all amoebozoan genomes published thus far; (2) the occurrence of RABL2 in *T. minutus* was remarkable because this gene was absent from the previously sequenced eustigmatophyte genomes (representing several species of the genus *Nannochloropsis*); and (3) the finding of RABL2 in jakobids and malawimonads, two deep eukaryotic lineages exhibiting many presumably primitive traits [36], supported the notion that RABL2 is an ancient eukaryotic gene. We therefore decided to carry out a detailed comparative evolutionary study of the RABL2 gene to address primarily the two following questions. Firstly, what is the phylogenetic distribution of this gene and when it originated during the evolution of eukaryotes? And secondly, does the functional association of RABL2 with the cilium in mammals reflect a situation in eukaryotes in general?

## Results and discussion

### RABL2 is a highly conserved small GTPase distinct from other Ras superfamily members

Taking advantage of the wealth of genomic and transcriptomic data that have recently become available for diverse eukaryotes, including data from our on-going genome and/or transcriptome sequencing projects, we assembled a large set of RABL2 sequences covering most of the eukaryote phylogenetic diversity (Additional file 1: Table S1). Careful manual curation was employed to ensure the highest possible quality of the sequence dataset, including completion of some sequences by targeted re-assembly of original sequencing reads or correction of wrong gene models deposited in databases (for technical details see the Methods section below and Additional file 1: Table S1). Our final dataset included RABL2 sequences from 118 species. During our searches we did not encounter a single case where we would be in doubts concerning the assignment of the sequence as a RABL2 ortholog or as an ortholog of another gene of the Ras superfamily. This indicates that RABL2 genes are highly conserved and do not tend to generate divergent lineage-specific paralogs (in-paralogs), which is in contrast to many other GTPases in the Ras superfamily [37, 38].

A note on nomenclature of RABL2 orthologs must be added here, as it has caused some confusion in the past. Different names have been used to denote RABL2 proteins, including Rab11B (*Trypanosoma brucei* RABL2, GenBank accession number AF234189.1), RabX3 (*T. brucei* RABL2 [39]), RabX32 (*Tetrahymena thermophila* RABL2 [40]), Rab_A50 (*Paramecium tetraurelia* RABL2 [40]), and RTW [38, 41]. The different nomenclature perhaps confused Lo et al. [29], who appear to have treated sequences denoted RABL2 and RTW as different groups (note also the aberrant topology of their tree presented in their Figure S2, suggesting that the “RTW” and “RABL2” sequences were not properly aligned to each other). Although the name RABL2 is not ideal (e.g., an unrelated GTPase – a true RAB family member – is labelled as “RabL2” in *Entamoeba histolytica* [42]), it is used in this paper to refer to all orthologs of the human RABL2A/RABL2B gene pair.

The assignment of all RABL2 orthologs was confirmed by a phylogenetic analysis including representative sequences of various Ras superfamily GTPases, which showed all annotated RABL2 genes as monophyletic with maximal statistical support (Additional file 2: Figure S1). We also tried to establish the phylogenetic position of the RABL2 branch among other lineages of the Ras superfamily. RABL2, together with several other Rab-like GTPases (IFT27, RJL, Spg1/Tem1), clearly belongs to the same subgroup as the traditional Rab, Ran, Ras, and Rho families, but no resolution among the many branches of this subgroup could be achieved and the relative branching order of the branches was highly sensitive to the substitution model employed and to the mask applied on the full alignment (Additional file 2: Figure S1 and data not shown). Rojas et al. [43] assumed that RABL2 is Metazoa-specific, and given clustering of RABL2 with RAN in their trees, they suggested that RABL2 emerged from duplication of RAN in the Metazoa lineage. While assuming the origin of RABL2 specifically in Metazoa is incorrect (see below), the common ancestry of RABL2 and RAN cannot be excluded.

### RABL2 proteins comprise a conserved GTPase domain and an extra C-terminal helix

**Fig. 1:**
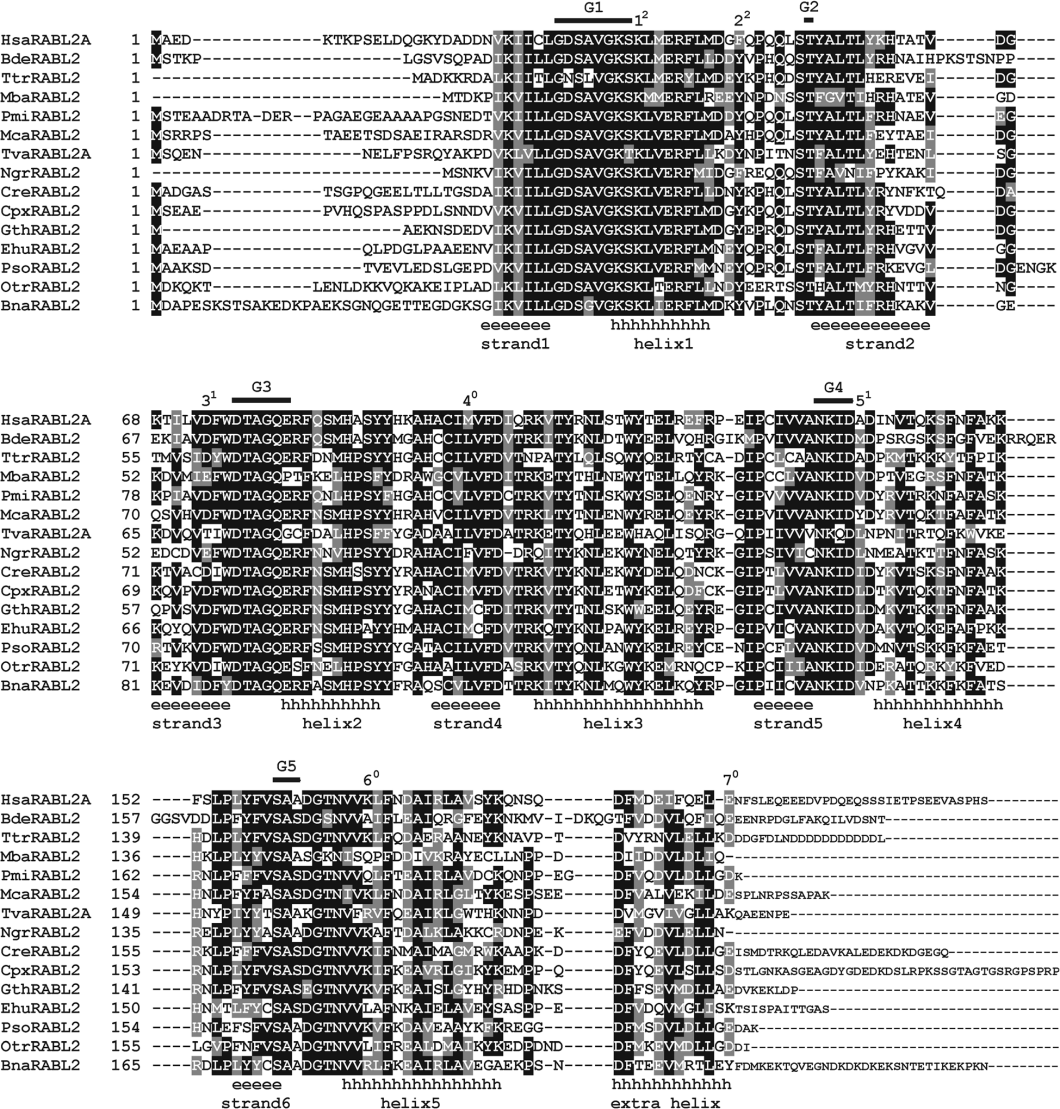
Annotated multiple alignment of representative RABL2 proteins sequences. The figure shows a subset of RABL2 sequences from a complete alignment provided as Additional file 3. The five conserved functionally important motifs of the Ras superfamily (G1 to G5 [45]) are marked on the top. Regions corresponding to secondary structure elements – α-helices and β-sheets – predicted for the RABL2 GTPase are indicated by series of letters “h” and “e”, respectively. The figure shows the prediction of α-helices and β-sheets as provided by PROMALS [113], but predictions using other tools were generally congruent with some differences in exact delimitation of the different elements. Note the predicted extra helix at the C-terminus that does not belong to the conserved core of a GTPase domain (comprised of the region from strand 1 to helix 5). The seven intron positions inferred to be ancestral for the RABL2 gene (see main text) are marked by consecutive numbers above the amino acid residues whose codon is located immediately upstream of the intron (phase 0) or is interrupted by the intron at the second or third position (phases 2 and 3). The phase of each intron is indicated by the number in superscript. The sequences of the extremely variable C-terminal tail encoded by the terminal ancestral exon (downstream of the 7^th^ ancestral intron) are not aligned, as no meaningful alignment can be produced for the sequences from different major eukaryotic groups. Species abbreviations used to label the RABL2 sequences: Hsa – *Homo sapiens*; Bde – *Batrachochytrium dendrobatidis*; Ttr – *Thecamonas trahens*; Mba – *Mastigamoeba balamuthi*; Pmi – *Planomonas micra*; Mca – *Malawimonas californiana*; Tva – *Trichomonas vaginalis*; Ngr – *Naegleria gruberi*; Cre – *Chlamydomonas reinhardtii*; Cpx – *Cyanophora paradoxa*; Gth – *Guillardia theta*; Ehu – *Emiliania huxleyi*; Pso – *Phytophthora sojae*; Otr – *Oxytricha trifallax*; Bna – *Bigelowiella natans*. Sequence identifiers are available in Additional file 1: Table S1

Multiple alignment of RABL2 sequences (Fig. 1; Additional file 3) shows a picture typical for many small GTPases. Whereas the central GTPases is highly conserved, the N- and C-termini show considerable variation in both length and sequence. Our massively expanded sampling confirms that RABL2 proteins lack a C-terminal prenylation motif, similar to other Rab-like GTPases and RAN (although it should be noted that the Rab-like GTPases do not constitute a phylogenetically coherent group – the absence of C-terminal prenylation is most likely a plesiomorphic state inherited from prokaryotic ancestors of the “Rab/Ran/Ras/Rho group” [44]). The most conserved and functionally important regions of the GTPase domain, called G1 to G5 regions [45], are very well conserved across all RABL2 sequences gathered here, indicating that all are functional GTPases. The G2 region, with includes a conserved threonine or less often serine residue that makes a hydrogen bond to an Mg^2+^ cation required for GTP hydrolysis, adopts a specific sequence pattern in RABL2 sequences, readily distinguishing them from other GTPases. While “TIG” is apparently ancestral and the most common motif in small GTPases (replaced by “TIE” in typical Ras family proteins and “TVF” in typical Rho family GTPases), RABL2 sequences typically feature the “T(Y/F)A” motif (very rarely modified to “TFG”, “THA”, or “TNA”; Additional file 3).

No three-dimensional structure has been solved yet for any RABL2 protein, but their high similarity to other small GTPases, especially Rabs, makes it likely that their tertiary structure and catalytic mechanism will be basically the same. We nevertheless used the broad multiple alignment of RABL2 sequences to predict the secondary structure of the proteins. While the boundaries of the various α-helices and β-strands and even prediction of the presence of some elements varied depending on a prediction tool employed, all tools agreed on the presence of a helix distal to the C-terminal helix of the canonical small GTPase structure (Fig. 1). Interestingly RAN GTPases also have a C-terminal extension with an extra helix [46, 47]. It is possible that the extra helices in RABL2 and RAN are homologous, which would support the specific phylogenetic relationship between these two GTPases suggested by some authors [43, 48]. Direct structural investigations of RABL2 proteins are needed to test this hypothesis and to determine the role of the C-terminal helix in the RABL2 functional cycle.

### RABL2 can be traced back to the last eukaryotic common ancestor (LECA)

**Fig. 2:**
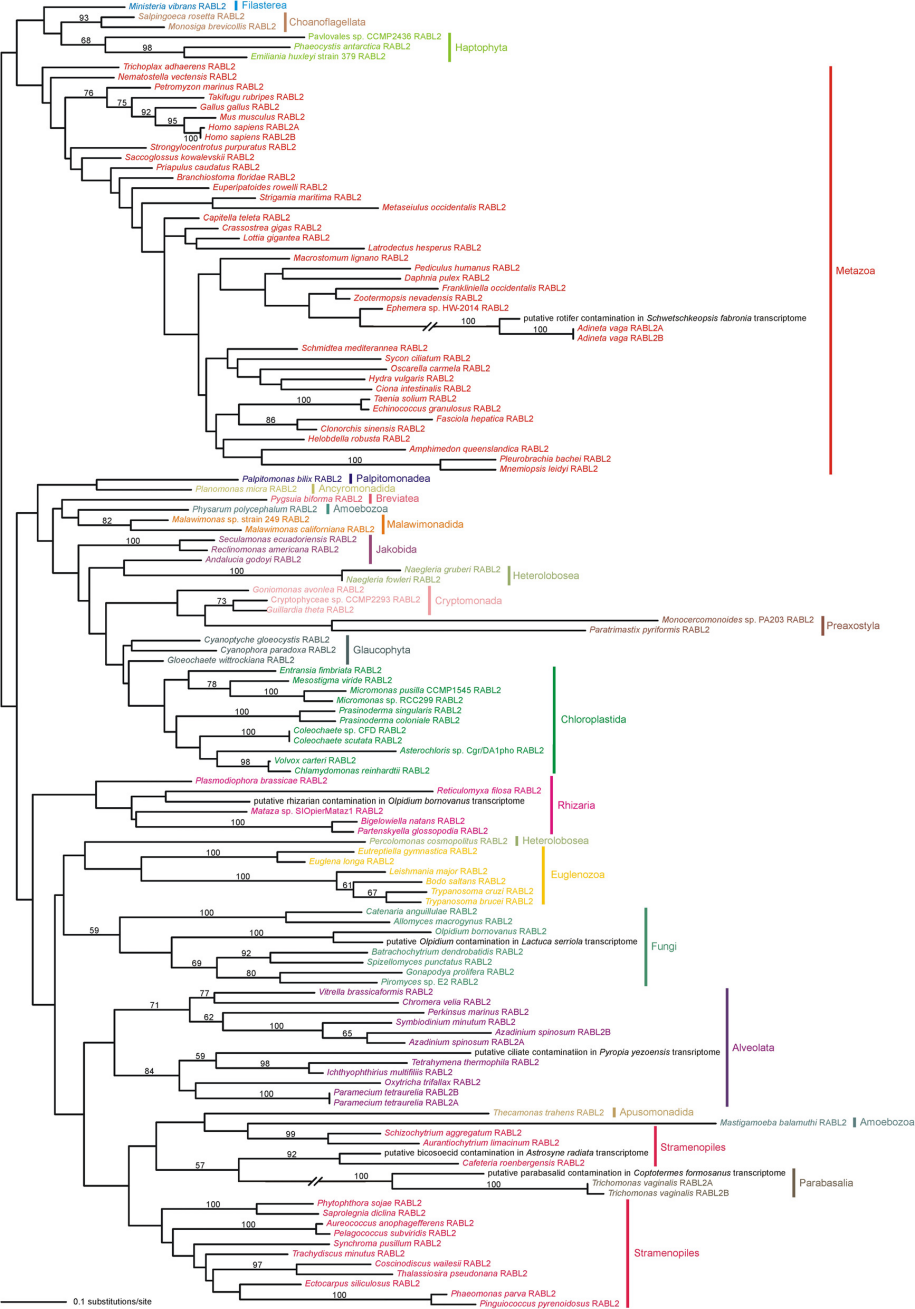
Maximum likelihood phylogenetic tree of RABL2 protein sequences. The tree was constructed from an alignment of complete or nearly complete RABL2 sequences (158 amino acid positions) using RAxML and the LG + Γ + F substitution model. Bootstrap support values are shown when higher than 50 %. Sequence identifiers are provided in Additional file 1: Table S1. Sequences representing the same major eukaryotic group (not necessarily monophyletic in the tree) are indicated with the same colour, sequences revealed as apparent contaminations (see main text and Additional file 2) are shown in black

We carried out a phylogenetic analysis of RABL2 sequences to investigate to what extent the evolution of RABL2 genes reflects species phylogeny and to check for a possible wrong classification of the source sequence data or contaminations. The phylogenetic signal in the short RABL2 sequences is necessarily limited and most branches in the resulting phylogenetic tree are thus unresolved (Fig. 2). However, strong statistical support was recovered for some unexpected relationships in the tree. For example, a RABL2 sequence coming from a cDNA library from the termite *Coptotermes formosanus* (GenBank accession number AFZ78866.1 [49]) is most closely related to the two RABL2 in-paralogs from the parabasalid *Trichomonas vaginalis* rather than clustering with sequences from the termite species *Zootermopsis nevadensis* (or at least other arthropods or metazoans). An obvious explanation is that this RABL2 sequence comes from one of the three different parabasalian symbionts known to reside in the gut of *C. formosanus* [50]. We identified a number of additional RABL2 sequences that represent obvious contamination; these are discussed in detail in Supplementary text in Additional file 2 (for their list see Additional file 1: Table S2).

**Fig. 3:**
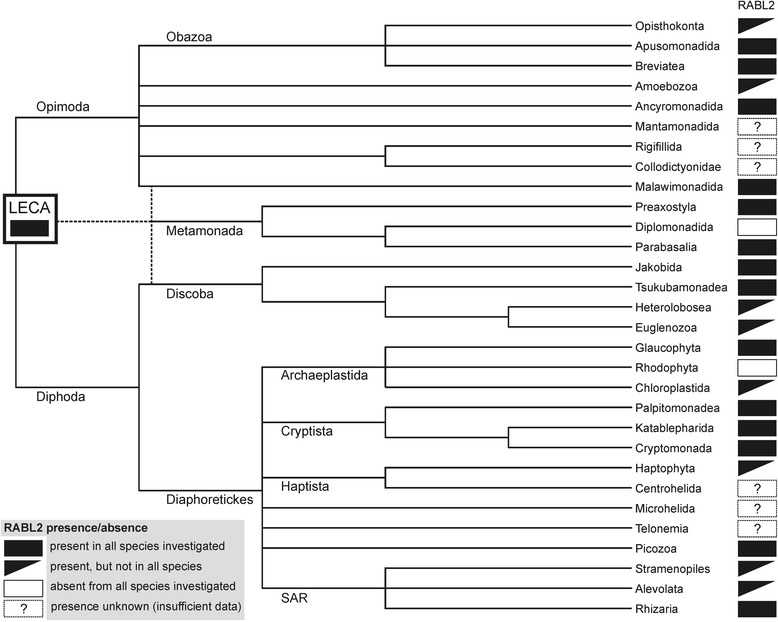
Occurrence of RABL2 genes in major eukaryotic lineages. The dendrogram showing the phylogenetic relationships among the taxa is drawn on the basis of current phylogenetic and phylogenomic literature. Multifurcations in the tree indicate lack of consensus on the topology in particular phylogenetic areas. The root of the tree is placed according to the most recent rooting hypothesis [53]. The position of Metamonada with respect to the root is unclear; sometimes they are placed sister to the group Discoba, while other analyses suggest metamonads may be sister to malawimonads or represent a deep group with unresolved affiliation. However, the unsettled position of metamonads, as well as alternative root positions suggested by other authors, do not change the inference on the occurrence of a RABL2 gene already in the LECA. For several eukaryotic lineages sufficiently complete genome or transcriptome data are still not available, so the presence or absence of RABL2 genes in them cannot be ascertained (indicated by the question marks)

Disregarding the contaminating sequences, the RABL2 tree is generally congruent with relationships among species and neither of the departures from the species tree topology received high bootstrap support. To more directly test for possible non-vertical inheritance of RABL2 genes in the eukaryote phylogeny, we used the approximately unbiased (AU) test [51] to compare the best RABL2 tree, inferred by the maximum likelihood (ML) method from an alignment excluding the identified contaminating sequences, with a tree constrained by the presumed tree topology (see Methods for details). The ‘species tree’ was not significantly worse than the best ML tree (*p* < 0.05), suggesting predominantly, if not purely, vertical inheritance of RABL2 genes. The RABL2 phyletic pattern can thus be readily interpreted as resulting from the presence of a RABL2 gene already in the LECA followed by its loss from some lineages descending from the LECA (Fig. 3). Indeed, we were able to detect RABL2 in at least some members of nearly all major eukaryotic lineages, including some small poorly studied groups with hitherto limited genomic data, including Breviatea, Apusomonadida, Malawimonadida, Ancyromonadida, Jakobida, Palpitomonadea, Glaucophyta, or Picozoa. Of the major eukaryotic lineages with sufficient amount of sequence data, only red algae (Rhodophyta) and diplomonads lack species with RABL2 genes, but the former case is easily explained by secondary loss due to loss of cilia in this group (see below) and the latter case may reflect the general reduction and divergence of diplomonad genomes. Missing data from a few small eukaryotic lineages, for instance Mantamonadida, Collodictyonidae, Telonemia, or Centrohelida, preclude a definite statement about the ancestral presence of RABL2 in the LECA, but since neither of the recently suggested positions of the root of the eukaryote phylogeny [52, 53] assumes that any of these minor lineages could be basal to those certainly possessing RABL2, we consider our inference concerning the presence of RABL2 in the LECA as very safe.

The phylogenetic analysis also confirms that existence of two RABL2 paralogs in a few species (namely *Homo sapiens*, the rotifer *Adineta vaga*, the parabasalid *Trichomonas vaginalis*, the ciliate *Paramecium tetraurelia*, and the dinoflagellate *Azadinium spinosum*) stems from very recent gene duplications (i.e., represents lineage-specific in-paralogs); in most cases the two paralogs are nearly identical at the protein sequence level. The list of species with the duplicated RABL2 genes is not surprising and generally reflects what is known about the dynamics of genome evolution in the respective lineages. Thus, whole genome duplication were described to have occurred in the lineages of *A. vaga* and *P. tetraurelia* [54, 55] and extensive gene duplications are known from the genomes of *T. vaginalis* and dinoflagellates [56, 57]. The duplication leading to the two paralogs in humans, traced back before the split between human and chimpanzee lineages but after the divergence of the Orangutan lineage [58], is thus somewhat singular in that it does not seem to passively reflect a general genome-specific evolutionary dynamics.

### The ancestral RABL2 gene consisted of at least eight exons, but the terminal exon has been repeatedly lost

The large number of manually curated exon-intron structures of RABL2 genes prompted us to investigate the evolution of the architecture of RABL2 genes. The number of introns in RABL2 genes ranges from 10 (in the cryptomonad *Guillardia theta*) to zero (Additional file 1: Table S1). Since the ancestral RABL2 is inferred to harbour multiple introns (see below), the intron-less RABL2 genes are a result of complete intron loss, which happened independently in at least eight lineages (discussed in detail in Supplementary text in Additional file 2).

We mapped the positions of introns in individual genes onto a multiple alignment of the respective protein sequences to identify homologous introns (Fig. 1; Additional file 4). Visual inspection of the resulting map revealed a clear phylogenetic signal in the pattern of intron positioning, as related species tend to exhibit similar exon-intron structures. For example, most metazoan RABL2 genes share seven conserved introns. Remarkably, comparison of the RABL2 introns across the whole span of eukaryote phylogeny revealed that these seven conserved metazoan introns are shared by a number of other eukaryotes on both sides of the eukaryotic root indicated by the most recent analyses [53]. This suggests that these introns (see Fig. 1 and Additional file 4) were most likely present already in the ancestral RABL2 gene resident in the genome of the LECA. Two more intron positions (see Additional file 4) are shared by several species across the Opimoda-Diphoda divide defined by Derelle et al. [53], but as discussed in detail in Supplementary text (Additional file 2), this is perhaps due to convergent intron gain. Therefore, we conservatively reconstruct the ancestral RABL2 architecture to have consisted of eight exons separated by seven introns.

There is no point in discussing all the cases of intron loss, gain, and sliding apparent from our intron map for the RABL2 gene (see Additional files 2 and 4), but one aspect is noteworthy. The terminal exon of the reconstructed ancestral RABL2 gene architecture is extremely variable in length and codes for a hypervariable C-terminal extension of the RABL2 GTPase (Fig. 1 and Additional file 4). Interestingly, it seems that this exon, and hence the C-terminal hypervariable extension, have been lost on multiple occasions by distantly related eukaryotes. This is most clearly apparent in the RABL2 genes from Pancrustacea (*Daphnia pulex* and insect genes in our sample), the amoebozoan *M. balamuthi*, the heterolobosean *Naegleria gruberi*, the two *Micromonas* strains in green algae, some stramenopiles, the ciliate *P. tetraurelia*, and the rhizarian *Reticulomyxa filosa*, which all lack an intron equivalent to the last (seventh) ancestral intron and the encoded protein sequence barely, if at all, extends beyond the position normally occupied by this intron (Fig. 1 and Additional file 4). Unfortunately, since the role of the hypervariable C-terminal extension of RABL2 proteins is unknown, the biological significance of the presumed frequent loss of the terminal exon remains unclear.

### At least 36 independent secondary losses of the RABL2 GTPase can be inferred based on the current sampling

Regardless the wide occurrence of RABL2 orthologs in eukaryotes, this GTPase is missing from a number of taxa, apparently due to multiple secondary losses. One actually needs to be cautious when claiming a gene absence in an organism, as this might be an artefact due to gaps in the respective genome assembly. We encountered such a case with the RABL2 gene from the cryptomonad *G. theta*. The gene sequence is absent from the published genome assembly (GenBank accession number AEIE00000000.1 [59]), but it has been recorded by transcriptome sequencing (Additional file 1: Table S1) and a full gene sequence could be assembled from genome sequencing reads for some reasons not integrated into the main genome assembly (data not shown). We similarly searched transcript data and original genomic reads for most species lacking a RABL2 in their genome sequence yet possessing closely related RABL2-containing species, and identified no additional case like *G. theta*. Nevertheless, we cannot exclude the possibility that some of the RABL2 absences considered here turn out to be such artefacts when the respective genome sequences are improved in the future.

**Fig. 4:**
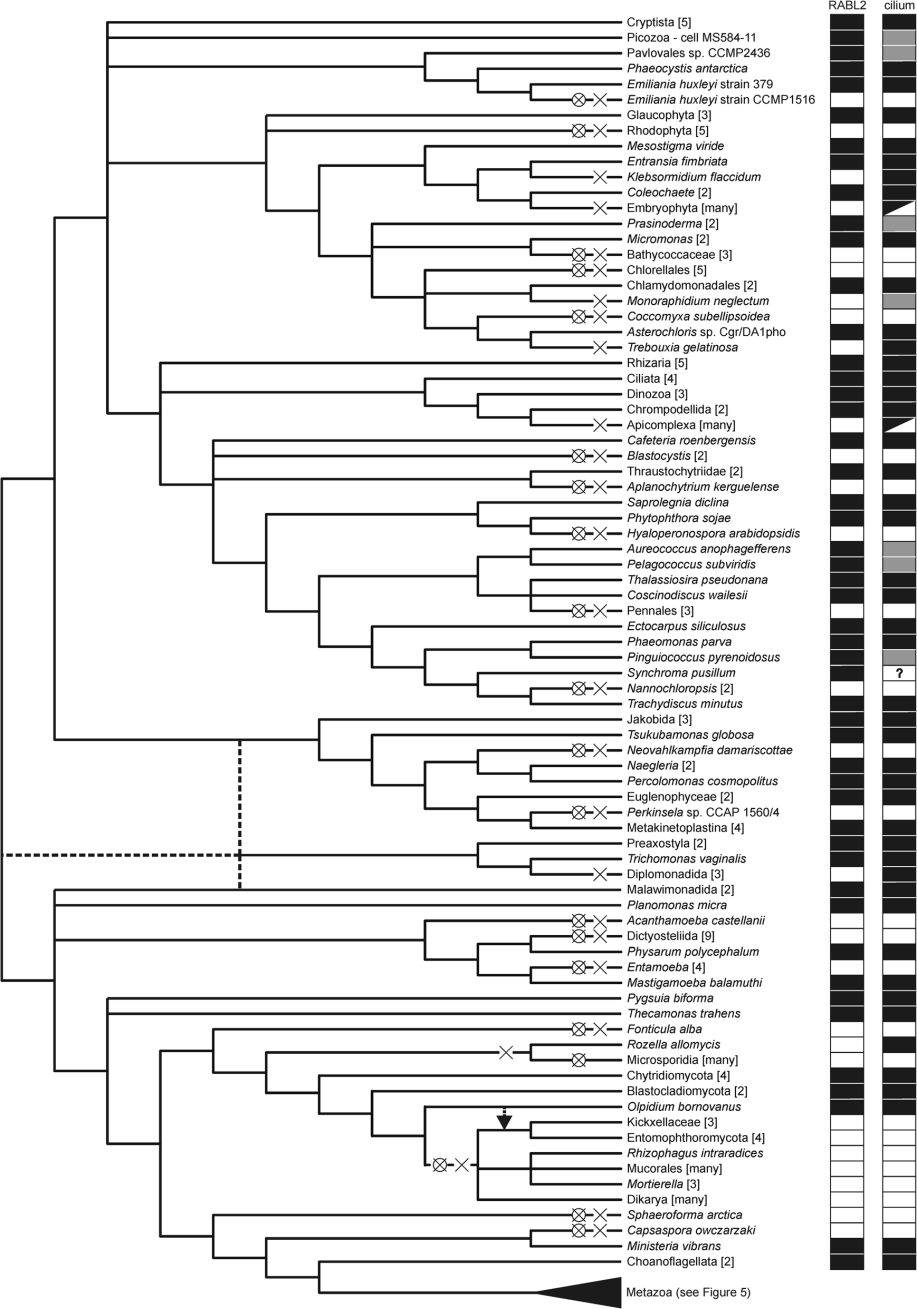
A fine-scale map of the phylogenetic distribution and losses of RABL2 genes in eukaryotes. The dendrogram indicating the relationships among the taxa was drawn with the same rationale as the one on Fig. 3. For each taxon the presence/absence of a RABL2 ortholog and of a cilium is indicated on the right (evidence for the presence of absence of a cilium for the different taxa is based an extensive literature survey complemented for some taxa with checking the presence of homologs of cilium-specific genes in their genome or transcriptome sequences). Well established (named) clades, where all species analyzed either possessed or lacked a RABL2 ortholog, were collapsed and displayed as a single terminal branch. The metazoan clade, which includes both RABL2-possessing and RABL2-lacking species, was also collapsed and is shown in detail in a separate scheme (Fig. 5). The number of the species representing the clade in our sample (see Additional file 1: Table S1 for their identity) is indicated in square brackets. The meaning of the symbols used for indicating the distribution and loss of RABL2 and the cilium is explained in Fig. 5. The position of the fungus *Olpidium bornovanus* is shown sister to all traditionally defined Eumycota (paraphyletic “Zygomycota” plus Dikarya) to conservatively indicate only a single unique loss of RABL2 in this group, but the dashed lines indicate that *Olpidium* may be specifically related to some “zygomycetes”, which would increase the number of RABL2 losses in Fungi (see main text for details)

**Fig. 5:**
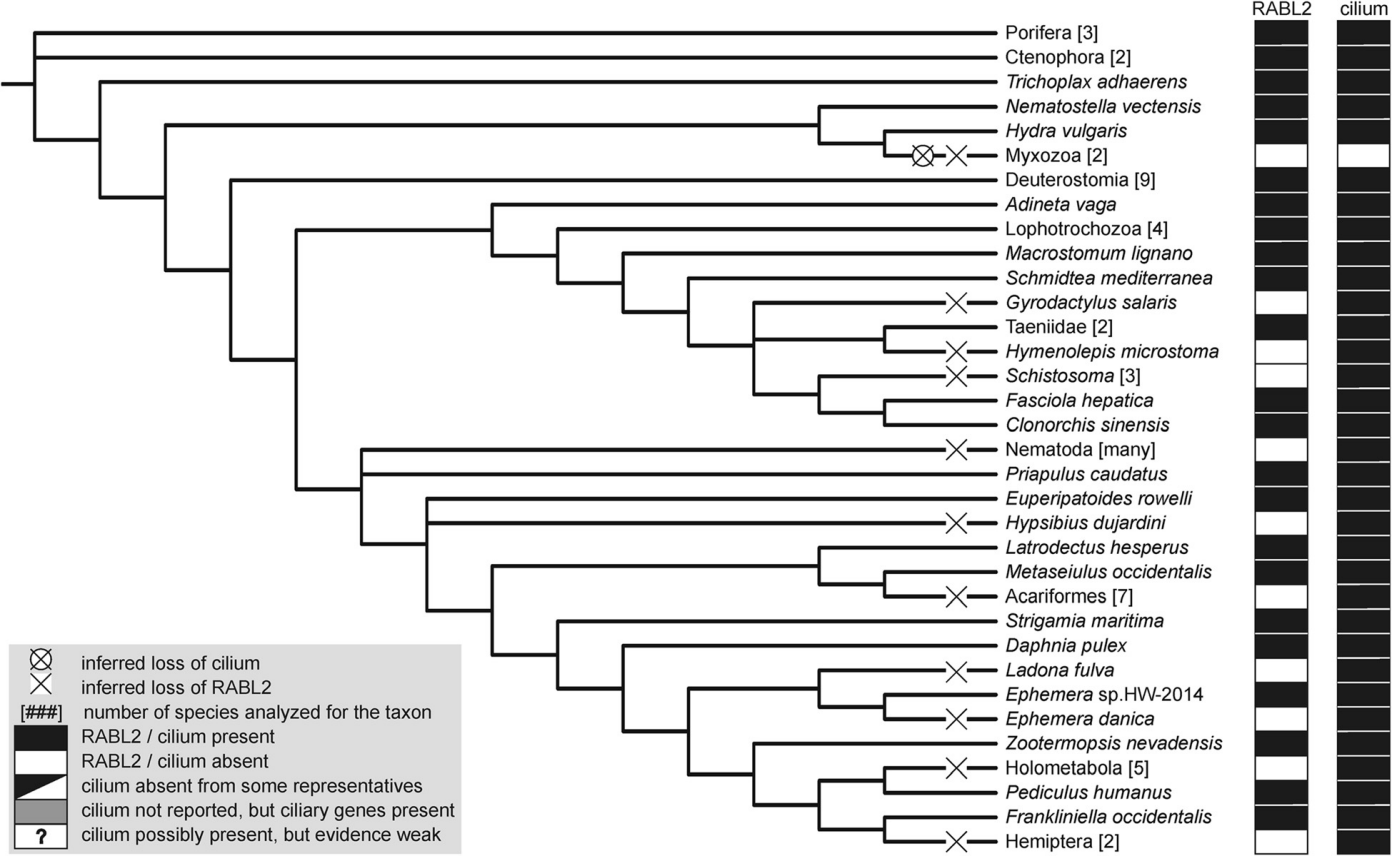
A fine-scale map of the phylogenetic distribution and losses of RABL2 genes in Metazoa. The figure was rendered using the same convention as Fig. 4

Given the lack of evidence for horizontal gene transfer (HGT) that would affect RABL2 genes (see above) and assuming that all the encountered absences are real, we need to invoke at least 36 independent losses of the RABL2 gene during the eukaryote evolution (Figs. 4 and 5). This number is perhaps a minimal estimate, not only because additional lineages lacking RABL2 independently on those currently known will likely be discovered with further sampling of eukaryote genomes, but also because we conservatively considered only a single RABL2 loss in “terrestrial fungi” (Eumycota), i.e., in the most speciose clade of fungi comprising the basal paraphyletic Zygomycota and the derived monophyletic Dikarya (Ascomycota and Basidiomycota) [60]. The uncertainty in this number stems from the fact that the genus *Olpidium*, traditionally classified as a “chytrid” owing to the presence of uniflagellated zoospores, is placed by molecular phylogenetic analyses among “zygomycetes”, although its exact position with respect to the different “zygomycete” lineages has not been resolved yet [60, 61]. It is, therefore, possible that the presence of RABL2 in *Olpidium*, but not in any “zygomycete” lineages represented by genome-sequenced representatives (Fig. 4; Additional file 1: Table S1), indicates more than one RABL2 losses in this phylogenetic area.

### RABL2 is missing from all cilium-lacking eukaryotes

The high number of independent RABL2 losses may be surprising, but a simple biological explanation exists for most of the loss events. Evidence discussed in Background shows that RABL2 is functionally associated with the cilium in those few species where it has been studied, and this association is apparently very strong, since our survey documents that RABL2 is missing whenever a species is known to lack the ability to construct cilia (Figs. 4 and 5). Indeed, despite performing near-exhaustive searches of available sequence data, we failed to find a single species that would represent a clear-cut case not obeying this rule. As explained in detail below, all candidate cases for the presence of a RABL2 gene in a cilium-lacking eukaryote have alternative biological explanations.

Let us discuss several cases that illustrate the correlation between the presence of a RABL2 gene and the ability to build a cilium. The very impetus to carry out this study was our observation that RABL2 is encoded by the genome of the amoebozoan *Mastigamoeba balamuthi*, while it is absent from a related lineage, *Entamoeba* (all *Entamoeb*a species with sequenced genomes). *M. balamuthi* and *Entamoeba* both belong to the anaerobic amoebozoan group Archaemoebae, but their immediately apparent difference is that the former is a free-living organism while the latter comprises endobiotic or parasitic species associated with various vertebrate hosts [62]. However, *M. balamuthi* (originally described as *Phreatamoeba balamuthi*), is also characterized by the presence of a single long anterior flagellum (cilium) [63], whereas the ciliary apparatus has been lost in the *Entamoeba* lineage [64]. Indeed, the only other amoebozoan presently known to harbour RABL2 is *Physarum polycephalum*, a plasmodial slime mould with biflagellated stages in its life cycle [65], whereas RABL2 is absent from all sequenced species of related cellular slime moulds, Dictyosteliida, lacking the ability to construct a cilium [64], as well as from *Acanthamoeba castellanii*, another cilium less amoebozoan with its genome sequence available (Fig. 4 and Additional file 1: Table S1).

Within diatoms, the basal paraphyletic grade of centric diatoms is characterized by the presence of flagellated sperm cells, whereas pennate diatoms (Pennales) have lost the ability to make flagellated stages [66]. In a nice correlation with this pattern we found RABL2 sequences in genome or transcriptome data from centric diatoms (Additional file 1: Table S1 and data not shown), whereas pennate diatoms lack RABL2, as could be tested by searching three complete genome sequences (Additional file 1: Table S1) and a number of deeply sequenced transcriptomes (see http://marinemicroeukaryotes.org/project_organisms for the list of species of pennate diatoms, i.e., the classes Bacillariophyceae and Fragilariophyceae, sequenced in the MMETSP project [67]). RABL2 sequences found in transcriptomic databases of two pennate diatom species result from contamination (see Additional file 1: Table S2 and Additional file 2). Another group of ochrophyte algae, eustigmatophytes, also includes species that differ in their capability of making a cilium, which correlates with the distribution of the RABL2 gene in this group. Thus, *T. minutus*, producing uniflagellated zoospores [68], does contain a RABL2 ortholog, whereas the species of the genus *Nannochloropsis* lack reported flagellated stages [69] and RABL2 (Fig. 4).

A striking recent instance of RABL2 loss has been encountered in the haptophyte alga *Emiliania huxleyi*. The RABL2 gene is absent from the published genome sequence of *E. huxleyi* strain CCMP1516 [70], even when raw sequencing reads from this strain are investigated, but we found partial RABL2 sequences in the EST data from *E. huxleyi* strain RCC1217. The latter strain is a haploid, flagellated form of the alga derived from a parental diploid, aflagellated strain RCC1216 [71]. Hence, the presence of the RABL2 transcripts in the EST survey of the haploid, but not the diploid, stage most likely means that the RABL2 gene is not transcribed in *E. huxleyi* cells when the cilium is absent. The lack of the RABL2 gene from the strain CCMP1516 then reflects the fact that this diploid, aflagellated strain was shown to have lost the ability to switch to the haploid stage, which is accompanied by the absence of numerous crucial ciliary genes from its genome [71, 72]. The loss of these genes, including RABL2, must be a relatively recent event, as the species *E. huxleyi* evolved only around 300,000 years ago [72]. We eventually found a complete RABL2 sequence in the transcriptome assembly for the *E. huxleyi* strain 379 (Additional file 1: Table S1), but the nature of this strain has not been reported yet; we predicted that it is a haploid stage or a mixture of diploid and haploid cells.

### The presence of RABL2 points to a cryptic flagellated stage in some species

Identification of RABL2 genes in some species may be surprising and deserves special attention. No flagellated stage was noticed upon the original description of the planktonic coccoid alga *Aureococcus anophagefferens* (Pelagophyceae) [73], hence the presence of a clear RABL2 ortholog in its genome seems to break the pattern discussed above. However, RABL2 is not without precedent, as homologs of a large number of cilium-associated genes have been previously found in this organism including, for example, a full complement of flagellar dyneins [74–77]. It was, therefore, suggested that *A. anophagefferens* most likely exhibits a cryptic flagellated stage [76, 77].

Our analyses revealed additional such candidates, hinted to by the presence of RABL2. Species of the genus *Prasinoderma* (*P. singularis* and *P. coloniale*), representing the poorly known green algal clade Prasinococcales, are known only as solitary or colonial non-motile walled coccoid cells, with no flagellated stages observed [78]. *Synchroma pusillum* is an amoeboid alga representing a recently erected class Synchromophyceae belonging to ochrophytes [79]. No flagellated stage was reported for any of the synchromophytes described to date, despite considerable attention paid to their life cycle [80]. *Pelagococcus subviridis* and *Pinguiococcus pyrenoidosus*, which belong to classes Pelagophyceae and Pinguiophyceae, respectively [81, 82], are marine planktonic cooccoid algae that both lack a reported flagellated stage [83, 84]. Yet all the five species listed above encode RABL2 homologs, as indicated by transcriptome data (Additional file 1: Table S1).

To gain a deeper insight into the significance of this observation, we probed the transcriptomes of these species with sequences of selected hallmark ciliary proteins (Additional file 1: Table S3). Indeed, all five species proved to express homologs of at least some ciliary genes, suggesting that all of them may have the capacity to form cilia. This would not be surprising at least for *Pelagococcus subviridis* and *Pinguiococcus pyrenoidosus*, since their close relatives (i.e., other pelagophytes or pinguiophytes) are known to produce zoospores or even are flagellates in their vegetative stage [82, 85, 86]. The least conclusive was the case of *Synchroma pusillum*, with only a few ciliary genes detected. While it is known that some typical ciliary genes may be conserved also in some cilium-lacking species [7], analyzing transcriptome data cannot provide a comprehensive view of the actual gene repertoire of the species, especially if the proportion of cells expressing the genes of interest is low, which might be the case of the putative ciliated cells of *S. pusillum*. It is also possible that *S. pusillum* lacks typical motile cilia (suggested by our failure to find the motor subunits of axonemal dyneins) and builds some sort of reduced immotile cilia with a specialized (e.g., sensory) function. Regardless, our identification of ciliary genes in *S. pusillum* and the other algae currently without known flagellated stages should provide an impetus for direct experimental investigation of possible cilia in these organisms.

### Some eukaryotes can assemble a cilium in the absence of RABL2

The analysis above establishes a pattern of RABL2 gene loss tightly associated with the loss of cilia in eukaryotes. However, the correlation is not perfect, since there are several taxa lacking RABL2 yet possessing a cilium (Figs. 4 and 5; Additional file 1: Table S1). Within Metazoa, these taxa include some arthropods and platyhelminths, the tardigrade *Hypsibius dujardini*, and all nematodes sequenced to date. Other RABL2-less ciliated eukaryotes include three groups of parasites: *Rozella allomycis* (an organism related to Microsporidia that produces uniflagellated zoospores [87]), diplomonads (represented here by *Giardia intestinalis* and two *Spironucleus* spp.), and apicomplexans with flagellated male gametes (*Plasmodium* spp. and Coccidia).

Somewhat surprising is the absence of a RABL2 gene from the recently released genome sequence of *Trebouxia gelatinosa*, a green alga (Trebouxiophyceae) known to produce ciliated zoospores [88] and related to the genus *Asterochloris* [89], including *Asterochloris* sp. Cgr/DA1pho here shown to harbour a typical RABL2 gene (Fig. 2 and Additional file 1: Table S1). No RABL2 gene could be identified even when we searched original genomic reads from *Trebouxia gelatinosa*, suggesting that the absence is authentic. Absence of a RABL2 ortholog from the draft genome assembly of the chlorophyte green alga *Monoraphidium neglectum* [90] is also notable. While the genus *Monoraphidium* is thought to lack flagellated stages [91], we found typical cilium-associated proteins, such as axonemal dyneins, to be encoded by the genome (data not shown). It is, therefore, possible that *M. neglectum* features a cryptic flagellated stage with cilia, yet built without the assistance of a RABL2 protein.

The remaining ciliated eukaryotes without RABL2 are found in streptophytes, a lineage comprised of land plants (embryophytes) and their closest green algal relatives [92]. Some embryophytes produce flagellated male gametes [93], but the two such representatives with sequenced genomes, the moss *Physcomitrella patens* and the lycophyte *Selaginella moellendorffii*, have no detectable RABL2. We additionally checked available transcriptome data from other ciliated embryophytes available in GenBank or the oneKP project (https://sites.google.com/a/ualberta.ca/onekp/; [94]), but no RABL2 ortholog could be found in any of them (except one case of apparent rotifer contamination in the transcriptome of the moss *Schwetschkeopsis fabronia*, see Fig. 2 and Additional file 2)*.* The streptophyte alga *Klebsormidium flaccidum* is known to produce flagellated zoospores [95] and although ciliary genes are not mentioned in the genome sequence report for the *K. flaccidum* strain NIES-2285 [96], they can be found in the genome sequence (data not shown). However, RABL2 appears to be absent from this genome assembly, and is missing also from the deeply sequenced transcriptomes from related species (*Klebsormidium subtile* [94], *Klebsormidium crenulatum* [97]). The apparent loss of RABL2 from *Klebsormidium* must be an independent event from the loss in embryophytes, because RABL2 orthologs are found in the klebsormidiophyte alga *Entransia fimbriata* and in the genus *Coleochaete*, which is one of the most closely related lineages to embryophytes [92].

It is at present difficult to provide a straightforward explanation for the absence of RABL2 in some ciliated species, as very limited functional information is available for RABL2 even for the single species where it was studied (i.e., mouse [29]). However, profiling the phylogenetic distribution of ciliary genes revealed that they often exhibit a pattern with recurrent absences in some ciliated taxa, e.g., some components of the IFT-A and IFT-B complexes and the BBSome [35] or the centriole/basal body [75]. In addition, it has been previously noted that cilia of some of the taxa here shown to lack RABL2 tend to be unusual or simplified compared to “prototypical” cilia of common model species. For example, the cilia of *Giardia intestinalis*, flagellated apicomplexans, and embryophytes were found to lack homologs of all or nearly all known proteins of transition zone complexes [98]. The absence of RABL2 from all sequenced nematodes may be related to the fact that this phylum exhibits only non-motile cilia with a highly simplified axoneme [99, 100]. Further functional characterization of RABL2 proteins from non-metazoan models (such as *Trypanosoma brucei* or *Chlamydomonas reinhardtii*) may help understand why RABL2 could have been lost from different flagellated eukaryotes.

## Conclusions

The phylogenetic breadth of the survey reported in this paper would be unimaginable a few years ago, but the current onslaught of genome and/or transcriptome data makes now possible to carry out analyses of the evolutionary history of individual genes that are nearly exhaustive when the level of the major branches of the eukaryote phylogeny is concerned. However, many gaps in our sampling still persist [101], and it would be interesting to investigate RABL2 genes is some groups missing from our sample. Specifically, having established that absence of a cilium implies absence of a RABL2 gene, we predict that many other cilium-lacking eukaryotic groups currently without reference genome sequences will prove to lack RABL2. Such candidate lineages include, for instance, centrohelids [102], zygnematophytes (conjugating green algae; [92]), or the aflagellated parabasalid *Dientamoeba fragilis* [103]. Future investigations of other presently ignored groups will help to pinpoint the dating of the already established RABL2 losses. For example, data from carpediemonads, free-living relatives of the parasitic diplomonads, would help answer the question whether the absence of RABL2 from diplomonads correlates with their parasitic lifestyle or whether it reflects an earlier loss that happened already in their free-living ancestor. The critical importance of sampling can further be demonstrated on streptophyte algae, where our previous survey of small GTPases based on a more restricted taxon sampling led to the conclusion that RABL2 (=RTW) was lost before the divergence of *Klebsormidium* and embryophytes [41], but our present finding of RABL2 in *Coleochaete* and *Entransia* revealed that RABL2 was lost independently from the *Klebsormidium* and the embryophyte lineages (Fig. 4). Thus, we predict that future studies with a still improved sampling will not only reveal additional lineages lacking RABL2, but will also show that some clades currently inferred to have lost RABL2 ancestrally actually include RABL2-containing species, which will increase the minimal required number of independent RABL2 losses well above the present 36 events.

Our study thus may have broader implications reaching beyond the field of small GTPases or the cilium research. A high number of independent loss events appear to have impacted the distribution of RABL2 genes in extant eukaryotes (Figs. 4 and 5) and also the architecture of the RABL2 genes themselves (see the loss of introns and the loss of the terminal exon in many RABL2 genes). This is a concrete manifestation of a somewhat neglected general phenomenon of reductive evolution [104], specifically recurrent reductive evolution [105], that has so far received much less attention than other evolutionary processes affecting organisms and their genomes, for instance gene family expansions or HGT, yet it may be a similarly significant factor shaping the extraordinary diversity of modern eukaryotes. We believe that examples like RABL2 will prompt the community of comparative genomicists to study recurrent gene loss in a more systematic fashion.

## Methods

### Assembling a reference set of RABL2 sequences

For the survey of RABL2 genes we tried to explore as many DNA and protein sequence resources as possible, including data in public databases as well as data from genome and/or transcriptome sequencing projects ongoing in our laboratories or in the labs of our collaborators (see Additional file 1: Table S1). The program BLAST and its variants (blastp, tblastn, blastn) [106], provided as on-line tools associated with particular public databases or in a stand-alone mode to search locally maintained databases, were used to identify RABL2 orthologs. Candidate hits were validated by reciprocal BLAST searches against our local database of annotated Ras superfamily GTPases to exclude orthologs of other GTPases. If no RABL2 ortholog was found in a predicted proteome available for the species, the respective genome sequence, and if available, transcriptome shotgun assemblies (TSA) or expressed sequence tags (ESTs), were checked by tblastn to find genes possibly skipped during the annotation of the genome. When needed, partial gene or transcript sequences were completed by iterative addition of matching raw Illumina or 454 reads in the Short sequence archive (SRA; http://www.ncbi.nlm.nih.gov/sra/). In a few cases a complete coding sequence was recorded in the genomic database, but the gene was fragmented into separate contigs or scaffolds due to gaps in intron regions. In such cases searching RAN-seq data in the SRA database helped to join the pieces to assemble a contiguous gene sequence (no effort was invested into filling in the remaining gaps in introns). Existing protein sequence predictions were carefully checked by considering transcript sequences of the same or closely related species (if available), inspecting a multiple alignment of RABL2 proteins sequences, and taking into account the existence of several broadly conserved intron positions. Protein sequence predictions that were apparently or likely incorrect were revised by manually redefining the exon-intron structure of the corresponding genes. A list of all sequences analyzed in this study, together with the corresponding accession numbers or sequence identifiers and source datasets, is available in Additional file 1: Table S1. All revised or newly predicted protein sequences are included in the multiple alignment available as Additional file 3. Sequences extracted from our unpublished genome or transcriptome assemblies were deposited at GenBank with accession numbers KU522217-KU522224.

### Phylogenetic analyses

The candidate RABL2 protein sequences were aligned using MAFFT (version 7, default parameters; http://mafft.cbrc.jp/alignment/server/ [107]) and the alignment was slightly adjusted manually. For the purpose of testing the assignment of the sequences as RABL2 orthologs and for an attempt to define the phylogenetic position of RABL2 in the Ras superfamily, the prealigned RABL2 sequences were added to an alignment of reference Rab, Ran, and IFT27 sequences built for a previous study [38]. This alignment already included some RABL2 sequences labelled as RTW (see above), which were used to guide combining the two alignments together. To include other possible relatives of RABL2, we also added selected reference sequences representing the Ras, Rho and RJL families, the less divergent N-terminal GTPase domain of several Miro proteins, several representatives of the poorly known, yet broadly conserved group of GTPase typified by the *Schizosaccharomyces pombe* protein Spg1, and some representatives of the recently defined Rup1 group of prokaryotic small GTPases (a likely outgroup of the eukaryotic sequences included [44]). The final alignment was masked to remove poorly conserved regions using the same mask as before [38], and a phylogenetic tree was inferred using the ML method as implemented in the program RAxML-HPC BlackBox (8.2.4) [108] accessible at the CIPRES Science Gateway (https://www.phylo.org/portal2; [109]). The substitution model employed was LG + Γ and branch support was assessed by the rapid bootstrapping algorithm that is an inherent part of the best tree search strategy of RAxML. The resulting tree is displayed as Additional file 2: Figure S1.

A separate phylogenetic analysis was performed for a set comprising only RABL2 sequences. The sequences (excluding the incomplete ones from *Roombia truncata*, *Tsukubamonas globosa*, and Picozoa sp.; Additional file 1: Table S1) were realigned using T-Coffee [110], masking all residues that have a consistency score below 8. The alignment was further processed using trimAl [111] to remove positions that had more than 20 % gaps and those belonging to a block of length <3 positions. A ML analysis was performed using RAxML (8.2) [108], by performing a search for the best ML tree combined with 100 bootstrap replicates (high-climbing algorithm) under the PROTGAMMALGF model. The resulting tree is displayed as Fig. 2. To test for possible non-vertical inheritance of RABL2 genes we employed the likelihood-based AU test [112] as follows. First, a set of RABL2 sequences excluding the putative contaminations (see Results and Discussion) was aligned and the alignment was processed as described above for the full RABL2 set. Next, best ML trees were calculated under the PROTGAMMALGF model using RAxML without a topological constraint and with an imposed constraint (a multifurcating tree) reflecting presumed relationships among RABL2 gene-possessing species (as displayed in Figs. 4 and 5). The unconstrained and constrained trees were then combined with 18 different trees randomly chosen from among bootstrap replicates, per site log-likelihoods were calculated for all 20 topologies using RAxML under the same model, and these values were compared using CONSEL [112].

### Other sequence analyses

The secondary structure of RABL2 proteins was predicted using several on-line tools, including PROMALS (http://prodata.swmed.edu/promals/promals.php; [113]), Jpred 4 (http://www.compbio.dundee.ac.uk/jpred4/index.html; [114]), and Quick2D (http://toolkit.tuebingen.mpg.de/quick2_d). The outputs of these tools were similar, so for the sake of simplicity only the prediction obtained using PROMALS is displayed in Fig. 1. A custom program written in the Java language was used to map the position of introns in individual RABL2 genes onto a multiple alignment of the respective protein sequences. The source of the information on intron positions was a manually curated dataset correcting many errors in gene models available in databases.

## Reviewers’ comments

### Reviewer’s report 1 (Berend Snel, Utrecht University, the Netherlands)

**Summary**: The authors offer a very thorough and detailed investigation of the evolution of RABL2 protein. The authors also clearly explain in the introduction that the general outline of the results in this paper where perhaps already somewhat known, which is admirable honesty. More and more similar studies both large scale as well as small scale such as this are currently appearing, but this one stands out for its attention to detail and solid analysis. As such I do not see any major objections to publishing this manuscript. However I do have some small points I would like to discuss.

Authors’ response*: Thank you for the positive judgement of our work.*

**Recommendations**: One small thing that is increasingly worrying me is that excellent analyses such as these could end up as “write only memory”, if the work here is not electronically applicable by future researchers. Specifically, if I now for another future bigger set of genomes want to identify RABL2 proteins, how can I use the information from this paper automatically? i.e., should the results not be summarized e.g., as a HMMER model from a curated alignment with a bitscore threshold that would find all RABL2 in all sequenced eukaryotic genomes? And should such a model be deposited in e.g., PANTHER?

Authors’ response: *The PANTHER database has the RABL2 group defined as “RAB-LIKE PROTEIN 2A-RELATED (PTHR24073:SF263)” (see*http://pantherdb.org/panther/family.do?clsAccession=PTHR24073:SF263*) and as we found out, it can readily classify even partial RABL2 sequences.*

On page 8 it is discussed that the pre-LECA origin (or outparalog) of RABL2 cannot be reliably inferred. One of the authors of this paper has been involved with the SCROLLSAW project that I think would be the preeminent tool to actually answer this question. Is this easily feasible for this question?

Authors’ response: *The reviewer is right that SCROLLSAW could in principle help define the phylogenetic position of the RABL2 lineage in the Ras superfamily, and in fact this was attempted in the previous study the reviewer is alluding to* [38] *(note that RABL2 genes are referred to as RTW in the cited paper). However, even focusing on the least divergent sequences representing individual ancient Ras superfamily paralogs (the very principle of SCROLLSAW) did not help to resolve this issue, as no statistical support was obtained for the deepest relationships among the paralogs (see Fig. 3 of the reference* [38]*). Resolving the early radiation of the Ras superfamily is perhaps beyond the limits of current methodology of phylogenetic inference.*

The manuscript is somewhat too long given the amount of results. For example perhaps the discussion on intron evolution (page 12/13) could be shortened, or less examples of striking concordant gene presence/absence patterns within linages between cilia and RABL2 (pages 15–17) could be given. Also the discussion on page 11 about (relative lack of) inparalogs, could also have been two/three sentences (first saying to be expected, second some examples of why/where this is to be expected, third pointing out that human is exception).

Authors’ response*: Although we believe that the original text deling with RABL2 inparalogs and with various examples of how RABL2 distribution correlates with the cilium distribution in different organismal groups was relevant, we shortened these parts a bit to make the manuscript more concise.*

With regards to the latter, I wonder if the very recent duplication in the hominid lineage could be related to sperm function (and positive selection?).

Authors’ response*: This is a very interesting and, in our view, a highly relevant idea, but testing it would necessitate a detailed comparative and functional analysis of RABL2 genes in hominids that is outside the focus of our present study.*

**Minor issues**: Page 2 (abstract): → structure apparently inherited → structure inherited, Page 20: silia → cilia.

Authors’ response*: both suggested modifications made (thank you for correcting the typo).*

### Reviewer’s report 2 (James O. McInerney, University of Manchester, United Kingdom)

**Summary**: This manuscript does three things very well, in my opinion. First of all, it shows the strong association between the presence of a gene RABL2 and the presence or absence of cilia in eukaryotes. Secondly, it notes that there are repeated losses of the gene throughout eukaryotic evolution - making the point in passing that we study gene loss far less often than we study gene gain. Finally, there is a nice observation on the repeated loss of the 8th exon in several genomes. The authors carry out extensive analyses of genomes across the diversity of eukaryotes and their manuscript is liberally sprinkled with observations and thoughts and warnings about the quality of genomic data.

Authors’ response*: Thank you a lot for these positive words about our paper.*

**Recommendations**: This is a very nice manuscript that has a lot of detail. The paper also makes the repeated comment that completed genomes are often a dangerous place to look for definitive evidence, particularly when you might be trying to conclude that a particular gene has been lost i a lineage. The paper does a really great job in defining the problem and outlining how you have approached it. I commend the authors for their care and attention. I notice that the authors have mentioned the tardigrade genome in their analysis - which assembly was used? There has been some considerable talk of Tardigrade genomes and I was wondering which of the assemblies was being used. Otherwise, I like this manuscript and commend the authors for producing such a thorough piece of work.

Authors’ response*: Many thanks again for the positive judgement of our work. The source of genomic data from the tardigrade (*Hypsibius dujardini*) used in our analysis is indicated in Additional file*1*: Table S1, which now also specifies the version of the genome assembly analyzed. We also checked the more recently released assembly reported for the same species by another research group (*[115]*; GenBank accession number LMYF00000000.1) and it also lacks a RABL2 ortholog, further supporting the absence of this gene from* H. dujardini*.*

**Minor issues**: none

## Additional files

Additional file 1: Table S1. The list of RABL2 genes analyzed. **Table S2.** RABL2 sequences identified as contaminations. **Table S3.** Ciliary genes in species with RABL2 yet unknown to have flagellated stages. (XLS 77 kb) Additional file 2:
**Supplementary text and Figure S1.** (PDF 1069 kb) Additional file 3:
**Multiple alignment of RABL2 sequences analyzed in this study.** The identity and taxonomic provenance of the sequences included is provided in Table S1 (authentic sequences) and S2 (contaminations) in Additional file 1. (FAS 61 kb) Additional file 4:
**Position and phase of introns in RABL2 genes mapped onto a multiple alignment of RABL2 protein sequences.** (HTML 59 kb)

## Abbreviations

AU test: Approximately unbiased test
EST: Expressed sequence tag
HGT: Horizontal gene transfer
ML: Maximum likelihood
TSA: Transcriptome shotgun assembly
