# Supplementary material for: A paneukaryotic genomic analysis of the small GTPase RABL2 underscores the significance of recurrent gene loss in eukaryote evolution
Source: Biol Direct. 2016 Feb 2;11:5. doi: 10.1186/s13062-016-0107-8 (PMC4736243; doi:10.1186/s13062-016-0107-8)
Supplement: Additional file 2: — Supplementary text and Figure S1. (PDF 1069 kb) [file 13062_2016_107_MOESM2_ESM.pdf]

Supplementary material (Additional file 2) for the paper

A paneukaryotic genomic analysis of the small GTPase RABL2 underscores the significance of recurrent gene loss in eukaryote evolution

Marek Eliáš[1]*, Vladimír Klimeš[1], Romain Derelle[2], Romana Petrželková[1], Jan Tachezy[3]

[1]University of Ostrava, Faculty of Science, Department of Biology and Ecology, Chittussiho 10, 710 00 Ostrava, Czech Republic

[2]Unité d'Ecologie, Systématique et Evolution, Centre National de la Recherche Scientifique UMR 8079, Université Paris-Sud, 91405 Orsay, France

[3]Charles University in Prague, Faculty of Science, Department of Parasitology, Viničná 7, 128 44 Prague 2, Czech Republic

*corresponding author

E-mail addresses of the authors:

ME: marek.elias@osu.cz

VK: thorin0@seznam.cz

RD: romain.derelle@gmail.com

RP: losssina@gmail.com

JT: jan.tachezy@natur.cuni.cz

This file includes Supplementary text (pp. 2-10) and Figure S1 (pp. 11-12).

Supplementary text

Contaminating RABL2 sequences in genomic and transcriptomic datasets

Our careful analyses of RABL2 orthologs is phylogenetically diverse eukaryotes revealed a number of cases where the presence of a RABL2 sequence in the respective transcriptomic dataset reflects contamination rather then genuine presence of a RABL2 gene in the target species. The identified cases are listed in Table S2 in Additional file 2. Identification of a RABL2 sequence in a cDNA library from the termite *Coptotermes formosanus* (AFZ78866.1) as contamination from a parabasalid is discussed in the main text. The rationale for identification of the remaining RABL2 sequences as contamination is provided below.

The pennate diatoms *Pseudo-nitzschia fraudulenta* (strain WWA7) and *Astrosyne radiata* (strain 13vi08-1A) are represented by transcriptomes sequenced as a part of the Marine Microbial Eukaryote Transcriptome Sequencing Project (MMETSP) [Keeling et al. 2014], and both transcriptome assemblies contain RABL2 sequences, although other pennate diatoms lack the gene. There are two somewhat different RABL2 versions in the *P. fraudulenta* dataset, but each is identical (at the nucleotide level) to one or another in-paralog in the dinoflagellate *Azadinium spinosum* (strain 3D9), which has also been sequenced in the MMETSP project. The authenticity of the two in-paralogs in *A. spinosum* is obvious from the phylogenetic analysis of RABL2 sequences, which places both sequences in a strongly supported clade with a RABL2 gene from another dinoflagellate, *Symbiodinium minutum* (Figure 2). Hence, we suspect that the RNA-seq library of *P. fraudulenta* was contaminated by the *A. spinosum* sample, presumably during the library preparation in the sequencing lab.

The RABL2 sequence found in the pennate diatom *A. radiata* dataset most likely comes from an unidentified bicosoecid, as the deduced protein sequence shares a unique insertion with a RABL2

protein from the bicosoecid *Cafeteria roenbergensis* (Additional file 3) and our phylogenetic analysis confirms that they are specifically related to the exclusion of other RABL2 sequences in the analysis (Figure 3). We checked the assembled transcriptome of *A. radiata* more closely and found other sequences probably coming from a bicosoecid. For example, the contig CAMNT_0004475253 codes for the protein actin, whose deduced sequence is most similar to the actin sequence previously reported from *C. roenbergensis* (ACR46932.1) among all protein sequences currently in GenBank. Meanwhile, actin apparently authentic for *A. radiata* is represented in the transcriptome by other contigs, e.g. CAMNT_0004479879 (the deduced protein is virtually identical to actin sequences from various diatoms). The most likely explanation is that the original culture of *A. radiata* was not monoeukaryotic, but contaminated by an unidentified bicosoecid species (the problem of such contaminations in the MMETSP transcriptomes was also noted previously [Keeling et al. 2014]).

A RABL2 sequence identified in the transcriptome assembly from the red alga *Pyropia yezoensis* (scaffold-ZULJ-2067957-Porphyra_yezoensis) generated by the OneKP project (https://sites.google.com/a/ualberta.ca/onekp/) is apparently contamination from a ciliate, as suggested by our phylogenetic analysis (Figure 2). Indeed, the independently generated genome sequence of the same species [Nakamura et al. 2013] lacks the corresponding gene, as do all other red algal genomes sequenced to date.

Checking the possible presence of RABL2 genes in embryophytes revealed only two candidates, a transcript contig from the moss *Schwetschkeopsis fabronia* (IGUH-2160775a-Schwetschkeopsis_fabronia) sequenced by the oneKP project (https://sites.google.com/a/ualberta.ca/onekp/; [Wickett et al. 2014]) and one contig from a transcriptome shotgun assembly from prickly lettuce (*L. serriola*; GenBank accession number

JO042926.1). However, our phylogenetic analysis (Figure 2) indicates that the sequence from the moss dataset is most closely related to RABL2 sequences from the rotifer *Adineta vaga*, so is most likely a contamination from a rotifer (not so unexpectedly, as rotifers are common microscopic inhabitants of mosses), while the sequence from the prickly lettuce transcriptome is most closely related to RABL2 from the fungus *Olpidium bornovanus* (Figure 2). The genus *Olpidium* comprises species parasitic for various plants [Sekimoto et al. 2011], so an obvious explanation for the presence of the RABL2 gene in the *L. serriola* transcriptome assembly is that the source plant material was infected by an *Olpidium*-related fungus. Indeed, we identified additional sequences in the transcriptome assembly that are most similar to sequences from *Olpidium* spp., including a contig representing the 28S rRNA gene exhibiting 99% identity to the 28S rRNA gene from *Olipidium brassicae* (data not shown).

Curiously, the transcriptome assembly of *Olpidium bornovanus* released by the Joint Genome Institute (http://genome.jgi.doe.gov/Olpbornscriptome/Olpbornscriptome.info.html) contains a single RABL2 sequence (Locus14751v1rpkm1.77) that is not represented in whole-genome reads available for the same species (the same isolate UBC F19785) in Sequence Read Archive (http://www.ncbi.nlm.nih.gov/sra). Our phylogenetic analysis shows this sequence to be specifically related to RABL2 genes from Rhizaria, whereas a different RABL2 sequence that we assembled from the genomic reads from *O. bornovanus* is related to other fungal RABL2 sequences (and the contaminating sequence in the transcriptome of *L. serriola*, see above; Figure 2). Searching the *O. bornovanus* transcriptome assembly identified contigs that represent 18S rRNA gene sequences from two different rhizarians, specifically cercozoans. One (e.g. the contig Locus7590v1rpkm5.67) is 99% identical to sequences in GenBank attributed to the genus *Gymnophrys*, actually representing the genus *Limnofila* (order Limnofilida in Cercozoa, see [Bass et al. 2009]). The other (e.g. the contig Locus13617v1rpkm2.01) is 99% identical to sequences representing the

environmental clade U in the cercozoan order Glissomonadida (see [Howe et al. 2011]). Hence, the RNA sample for the transcriptome sequencing was likely obtained from a *O. bornovanus* culture contaminated by two different cercozoans, one of which is the source of the RABL2 sequence Locus14751v1rpkm1.77 (while the authentic *O. bornovanus* identified in the genomic reads is not represented in the assembled transcriptome).

Finally, complete or partial RABL2 sequences could be identified in transcriptome data from from some holometabolous insects (Table S2 in Additional file 1), despite the fact that RABL2 genes are absent from all genome sequences obtained so far for members of this group. Although we did not include these sequences into the phylogenetic analysis presented as Figure 2, BLAST searches revealed that all these sequences are obvious contaminations, most often from trypanosomatids (common inhabitants of the insect gut).

Additional discussion on the evolution of exon-intron structure of RABL2 genes

As mentioned in the main text, while the RABL2 gene in the LECA posessed multiple introns, some RABL2 genes in extant species are not interrupted by introns, implying complete intron loss. As evident from the list in Table S1 (Additional file 1), complete intron loss most likely occurred independently in the lineages leading to the following eight taxa: the calcarean sponge *Sycon ciliatum*, the apusomonad *Thecamonas trahens*, the ancyromond *Planomonas micra*, the parabasalid *Trichomonas vaginalis*, kinetoplastids (or more precisely, a clade comprising at least eubodonoids plus trypanosomatids), tha jakobid *Andalucia godoyi*, labyrinthuleans (or more precisely, a clade of the thraustochytrids *Aurantiochytrium* and *Schizochytrium*), and the diatom *Thalassiosira pseudonana.* Many of these taxa are know to have a low density of spliceosomal introns in their genomes in general, so the absence of introns in their RABL2 genes is not particularly striking.

Some instructive lessons about the evolution of exon-intron structure of RABL2 genes are provided by the well-sampled metazoan genes. While they generally share seven conserved introns, variation to this structure is introduced primarily by losses of some of these introns in some metazoans, down to no intron being present in the RABL2 gene in the sponge *S. ciliatum* (Table S1 in Additional file 1). In the RABL2 gene of *Daphnia pulex* one of the introns has changed phase by moving by one nucleotide towards the 5'-end – a case of the so-called intron sliding [Rogozin et al. 2012]. The two RABL2 paralogs in the rotifer *A. vaga* share an intron at a new position not occupied by an intron in any eukaryote sampled to date – an apparent case of intron gain.

Strikingly, the RABL2 genes from the ctenophores (*Mnemiopsis leidyi* and *Pleurobrachia bachei*) depart completely from the metazoan RABL2 gene structure, as neither of their three introns are homologous to introns in other metazoans (Additional file 4). This unusual exon-intron structure cannot be explained by the presumed basal position of ctenophores in the metazoan phylogeny [Whelan et al. 2015], because all seven conserved metazoan RABL2 introns appear to correspond to introns present in the RABL2 gene of the LECA (see the main text). The third intron in the ctenophore RABL2 genes is unique for this group, while the the first and second are positionally equivalent to introns found in the filasterean *Ministeria vibrans* and the first is furthermore positionally equivalent to an intron in the RABL2 genes from cryptomonads (*G. theta* and the unidentified species Cryptophyceae sp. CCMP2293; Additional file 4). The large phylogenetic distance beween ctenophores and filastereans on the one side and cryptomonads on the other suggest that convergent intron gain is the most likely explanation for the single shared intron position. However, Filasterea are phylogenetically rather close to Metazoa (specifically, it is a lineage sister to a clade of Metazoa plus choanoflagellates [Torruella et al. 2015]), so we considered a possibility that the two intron positions shared by RABL2 genes in *Ministeria* and ctenophores reflect their specific relationship. Excluding horizontal gene transfer, another possible scenario is

hidden paralogy, i.e. assuming RABL2 duplication in an ancestor of Filasterea and metazoans, reorganization of the exon-intron structure in one paralog, and differential loss of the two paralogs from Filasterea and ctenophores on the one side and other metazoans and choanoflagellates on the other side. However, phylogenetic analyses of RABL2 sequences do not suggest specific relationship between the RABL2 genes in *Ministeria* and ctenophores (Figure 2 and data not shown). Another explanation is homoplasy, coming in two different versions. The first is covergent gain independently in the ctenophore and *Ministeria* lineages, whereas the second assumes gain of the two novel introns in an ancestor of Filasterea and metazoans followed by their parallel loss in choanoflagellates and in Metazoa after the spit of the ctenophore lineage. We favour independent gains, as this view is supported by a recent study of exon-intron structures of genes in the ctenophore *M. leidyi*, which showed that they tend to generally depart from the structures found in other metazoans [Lehmann et al. 2013], suggesting some large-scale turnover of introns in the ctenophore lineage.

One more intron position shared by RABL2 genes coming from species across the Opimoda-Diphoda divide [Derelle et al. 2015] is found in RABL2 genes from Chytridiomycota, the cryptomonad *G. theta*, and a subset of stramenopiles (Additional file 4). Again, convergent intron gain is the most likely explanation, because assuming intron homology in this case (and hence the presence of the respective intron in the RABL2 gene in the LECA) would necessitate a high number of independent intron losses. This expanation is further supported by the fact that the RABL2 genes in both *G. theta* and Chytridiomycota exhibit signatures of extensive lineage-specific reorganization of the exon-intron structure. The RABL2 genes in Chytridiomycota have not retained a single out of the seven confidently inferred ancestral introns, while they have apparently gained novel introns not seen in other groups (Additional file 4). Similarly, the RABL2 gene in *G. theta* exhibits only two of the ancestral introns, while at least five novel introns have been apparently gained in the

cryptomonad lineage (Additional file 4).

An interesting issue that also deserves to be mentioned concerns a intron apparently shared by RABL2 genes of the following subset of taxa: the green algae *Chlamydomonas reinhardtii* and *Asterochloris* sp. Cgr/DA1pho, the cryptomonads *Guillardia theta* and Cryptophyceae sp. CCMP2293, the haptophytes *Emiliania huxleyi* and *Phaeocystis antarctica*, and the stramenopiles *Phytophthora sojae*, *Ectocarpus siliculosus*, and *Trachydiscus minutus*. This intron occupies a position just a few nucleotides downstream of a position corresponding to the 5th intron of the reconstructed ancestral RABL2 gene architecture, and when present, the 5th ancestral intron is missing from the gene (Additional file 4). Organisms with this intron pattern are not directly related and are in fact intermingled with taxa that exhibit the ancestral state (i.e. the glaucophyte *Cyanophora paradoxa*, the cryptomonad *Goniomonas avonlea*, and alveolates and rhizarians). As with the situation encountered in ctenophores and *Ministeria* discussed above, one may hypothesize hidden paralogy or homoplasy as possible scenarios to explain the alternating intron patterns in these groups. We do not see any evidence for the "hidden paralogy" scenario in the phylogenetic tree of RABL2 genes, and while the possibility of multiple independent gains of the new intron accompanied by the loss of the nearby 5th ancestral intron cannot be ruled out, we would promote a third a scenario as the most realistic in this case. Hence, we posit that an ancestor of all these lineages, perhaps an ancestor of the whole Diaphoretickes clade, gained the novel intron downstream of the 5th ancestral intron, but this configuration was evolutionarily unstable, because this created a very short exon (only 11 bp long) possibly decreasing the accuracy of splicing. This was resolved by differential loss by one or the other intron in different lineages of Diaphoretickes. Investigating RABL2 gene structures in additional Diaphoretickes linegase, such as Palpitomonadea or Telonemia, may help to decide which of the possible scenarios is more likely.

References to the supplementary text

Bass D, Chao EE, Nikolaev S, Yabuki A, Ishida K, Berney C, Pakzad U, Wylezich C, Cavalier-Smith T. Phylogeny of novel naked Filose and Reticulose Cercozoa: Granofilosea cl. n. and Proteomyxidea revised. Protist. 2009;160:75-109. doi:10.1016/j.protis.2008.07.002.

Derelle R, Torruella G, Klimeš V, Brinkmann H, Kim E, Vlček Č, Lang BF, Eliáš M. Bacterial proteins pinpoint a single eukaryotic root. Proc Natl Acad Sci U S A. 2015;112:E693-E699.doi: 10.1073/pnas.1420657112.

Howe AT, Bass D, Chao EE, Cavalier-Smith T. New genera, species, and improved phylogeny of Glissomonadida (Cercozoa). Protist. 2011;162:710-722. doi:10.1016/j.protis.2011.06.002.

Keeling PJ, Burki F, Wilcox HM, Allam B, Allen EE, Amaral-Zettler LA, Armbrust EV, Archibald JM, Bharti AK, Bell CJ et al. The Marine Microbial Eukaryote Transcriptome Sequencing Project (MMETSP): illuminating the functional diversity of eukaryotic life in the oceans through transcriptome sequencing. PLoS Biol. 2014;12:e1001889. doi:10.1371/journal.pbio.1001889.

Lehmann J, Stadler PF, Krauss V. Near intron pairs and the metazoan tree. Mol Phylogenet Evol. 2013;66:811-823. doi:10.1016/j.ympev.2012.11.012.

Nakamura Y, Sasaki N, Kobayashi M, Ojima N, Yasuike M, Shigenobu Y, Satomi M, Fukuma Y, Shiwaku K, Tsujimoto A, Kobayashi T, Nakayama I, Ito F, Nakajima K, Sano M, Wada T, Kuhara S, Inouye K, Gojobori T, Ikeo K. The first symbiont-free genome sequence of marine red alga, Susabi-nori (*Pyropia yezoensis*). PLoS One. 2013;8(3):e57122. doi:10.1371/journal.pone.0057122.

Rogozin IB, Carmel L, Csuros M, Koonin EV. Origin and evolution of spliceosomal introns. Biol Direct. 2012;7:11. doi:10.1186/1745-6150-7-11.

Sekimoto S, Rochon D, Long JE, Dee JM, Berbee ML. A multigene phylogeny of *Olpidium* and its implications for early fungal evolution. BMC Evol Biol. 2011;11:331. doi:10.1186/1471-

2148-11-331.

Torruella G, de Mendoza A, Grau-Bové X, Antó M, Chaplin MA, Del Campo J, Eme L, Pérez-Cordón G, Whipps CM, Nichols KM, Paley R, Roger AJ, Sitjà-Bobadilla A, Donachie S, Ruiz-Trillo I. Phylogenomics Reveals Convergent Evolution of Lifestyles in Close Relatives of Animals and Fungi. Curr Biol. 2015;25:2404-2410. doi:10.1016/j.cub.2015.07.053.

Whelan NV, Kocot KM, Moroz LL, Halanych KM. Error, signal, and the placement of Ctenophora sister to all other animals. Proc Natl Acad Sci U S A. 2015 ;112:5773-8. doi:10.1073/pnas.1503453112.

Wickett NJ, Mirarab S, Nguyen N, Warnow T, Carpenter E, Matasci N, Ayyampalayam S, Barker MS, Burleigh JG, Gitzendanner MA et al. Phylotranscriptomic analysis of the origin and early diversification of land plants. Proc Natl Acad Sci U S A. 2014;111:E4859-4868. doi:10.1073/pnas.1323926111.

Wuichet K, Søgaard-Andersen L. Evolution and diversity of the ras superfamily of small GTPases in prokaryotes. Genome Biol Evol. 2014;7:57-70. doi:10.1093/gbe/evu264.

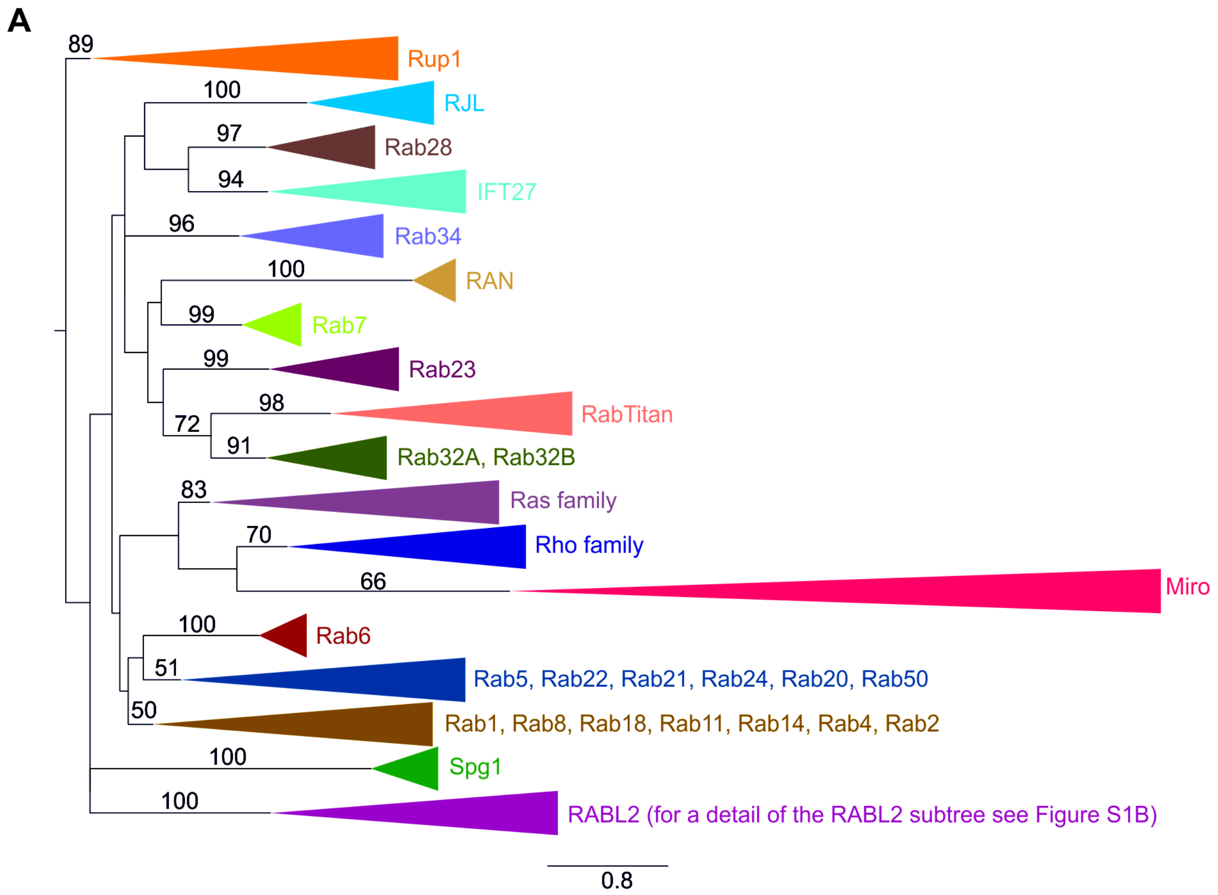

Figure S1. RABL2 is a robustly delimited subgroup of the Ras GTPase superfamily. The tree was inferred using RAxML (LG+Γ+F substitution model) using an alignment of all RABL2 seuqences analyzed in this study and a selection of sequences representing other major subgroups of the "Rab/Ran/Ras/Rho group", including the prokaryotic subgroup Rup1 [Wuichet and Søgaard-Andersen 2014]. Boostrap values are shown for branches if ≥ 50%, the scale bars indicate the number of substitutions per site. **A)** A full tree with the major groups of GTPases collapsed for simplicity. **B) next page** – A detail of the clade comprising RABL2 sequences (accession numbers available in Table S1 in Additional file 1).

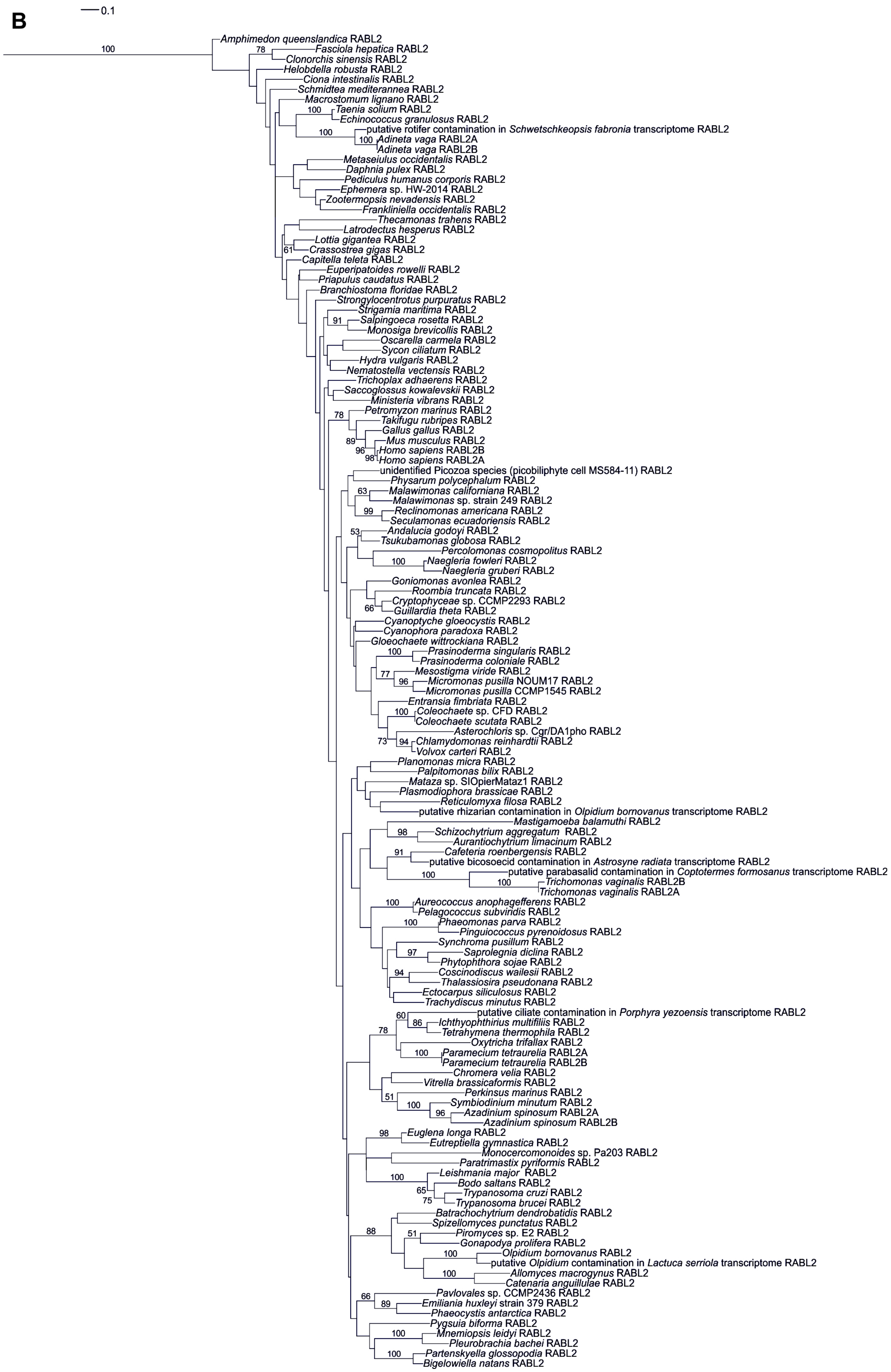
B
0.1
Amphimedon queenslandica RABL2
Fasciola hepatica RABL2
Clonorchis sinensis RABL2
Helobdella robusta RABL2
Ciona intestinalis RABL2
Schmidtea mediterannea RABL2
Macrostomum lignano RABL2
Taenia solium RABL2
Echinococcus granulosus RABL2
putative rotifer contamination in Schwetschkeopsis fabronia transcriptome RABL2
Adineta vaga RABL2A
Adineta vaga RABL2B
Metaseiulus occidentalis RABL2
Daphnia pulex RABL2
Pediculus humanus corporis RABL2
Ephemera sp. HW-2014 RABL2
Zootermopsis nevadensis RABL2
Frankliniella occidentalis RABL2
Thecamonas trahens RABL2
Latrodectus hesperus RABL2
Lottia gigantea RABL2
Crassostrea gigas RABL2
Capitella teleta RABL2
Euperipatoides rowelli RABL2
Priapulus caudatus RABL2
Branchiostoma floridae RABL2
Strongylocentrotus purpuratus RABL2
Strigamia maritima RABL2
Salpingoeca rosetta RABL2
Monosiga brevicollis RABL2
Oscarella carmela RABL2
Sycon ciliatum RABL2
Hydra vulgaris RABL2
Nematostella vectensis RABL2
Trichoplax adhaerens RABL2
Saccoglossus kowalevskii RABL2
Ministeria vibrans RABL2
Petromyzon marinus RABL2
Takifugu rubripes RABL2
Gallus gallus RABL2
Mus musculus RABL2
Homo sapiens RABL2B
Homo sapiens RABL2A
unidentified Picozoa species (picobiliphyte cell MS584-11) RABL2
Physarum polycephalum RABL2
Malawimonas californiana RABL2
Malawimonas sp. strain 249 RABL2
Reclinomonas americana RABL2
Seculamonas ecuadoriensis RABL2
Andalucia godoyi RABL2
Tsukubamonas globosa RABL2
Percolomonas cosmopolitus RABL2
Naegleria fowleri RABL2
Naegleria gruberi RABL2
Goniomonas avonlea RABL2
Roombia truncata RABL2
Cryptophyceae sp. CCMP2293 RABL2
Guillardia theta RABL2
Cyanoptyche gloeocystis RABL2
Cyanophora paradoxa RABL2
Gloeochaete wittrockiana RABL2
Prasinoderma singularis RABL2
Prasinoderma coloniale RABL2
Mesostigma viride RABL2
Micromonas pusilla NOUM17 RABL2
Micromonas pusilla CCMP1545 RABL2
Entransia fimbriata RABL2
Coleochaete sp. CFD RABL2
Coleochaete scutata RABL2
Asterochloris sp. Cgr/DA1pho RABL2
Chlamydomonas reinhardtii RABL2
Volvox carteri RABL2
Planomonas micra RABL2
Palpitomonas bilix RABL2
Mataza sp. SIOpierMataz1 RABL2
Plasmodiophora brassicae RABL2
Reticulomyxa filosa RABL2
putative rhizarian contamination in Olpidium bornovanus transcriptome RABL2
Mastigamoeba balamuthi RABL2
Schizochytrium aggregatum RABL2
Aurantiochytrium limacinum RABL2
Cafeteria roenbergensis RABL2
putative bicosoecid contamination in Astrosyne radiata transcriptome RABL2
putative parabasalid contamination in Coptotermes formosanus transcriptome RABL2
Trichomonas vaginalis RABL2B
Trichomonas vaginalis RABL2A
Aureococcus anophagefferens RABL2
Pelagococcus subviridis RABL2
Phaeomonas parva RABL2
Pinguiococcus pyrenoidosus RABL2
Synchroma pusillum RABL2
Saprolegnia diclina RABL2
Phytophthora sojae RABL2
Coscinodiscus wailesii RABL2
Thalassiosira pseudonana RABL2
Ectocarpus siliculosus RABL2
Trachydiscus minutus RABL2
putative ciliate contamination in Porphyra yezoensis transcriptome RABL2
Ichthyophthirius multifiliis RABL2
Tetrahymena thermophila RABL2
Oxytricha trifallax RABL2
Paramecium tetraurelia RABL2A
Paramecium tetraurelia RABL2B
Chromera velia RABL2
Vitrella brassicaformis RABL2
Perkinsus marinus RABL2
Symbiodinium minutum RABL2
Azadinium spinosum RABL2A
Azadinium spinosum RABL2B
Euglena longa RABL2
Eutreptiella gymnastica RABL2
Monocercomonoides sp. Pa203 RABL2
Paratrimastix pyriformis RABL2
Leishmania major RABL2
Bodo saltans RABL2
Trypanosoma cruzi RABL2
Trypanosoma brucei RABL2
Batrachochytrium dendrobatidis RABL2
Spizellomyces punctatus RABL2
Piromyces sp. E2 RABL2
Gonapodya prolifera RABL2
Olpidium bornovanus RABL2
putative Olpidium contamination in Lactuca serriola transcriptome RABL2
Allomyces macrogynus RABL2
Catenaria anguillulae RABL2
Pavlovales sp. CCMP2436 RABL2
Emiliania huxleyi strain 379 RABL2
Phaeocystis antarctica RABL2
Pygsuia biforma RABL2
Mnemiopsis leidyi RABL2
Pleurobrachia bachei RABL2
Partenskyella glossopodia RABL2
Bigelowiella natans RABL2
