# Supplementary material for: A paneukaryotic genomic analysis of the small GTPase RABL2 underscores the significance of recurrent gene loss in eukaryote evolution
Source: Biol Direct. 2016 Feb 2;11:5. doi: 10.1186/s13062-016-0107-8 (PMC4736243; doi:10.1186/s13062-016-0107-8)
Supplement: Additional file 4: — Position and phase of introns in RABL2 genes mapped onto a multiple alignment of RABL2 protein sequences. (HTML 59 kb) [file 13062_2016_107_MOESM4_ESM.html]

Additional file 4

**Additional file 4: Position and phase of introns in RABL2 genes mapped onto a multiple alignment of RABL2 protein sequences.**  
The intron positions are marked by highlighting amino acid residues whose codon is located immediately upstream of the intron (phase 0; in red) or is interrupted by the intron at the second or third position (phases 2 and 3; in
green or blue, respectively). The seven intron positions inferred to have been present in the RABL2 gene in the LECA (see main text) are numbered on the top of the alignment. Note that genes with the coding sequence in a single
exon, and sequences represented only by transcript data, are not included in the alignment. The identity and taxonomic provenance of the sequences included is provided in Table S1 in Addditional file 1.

```
                                                                            1         2                                           3                         4                                      5                                                           6                                                     7
HsaRABL2A -MAED------------------------------KTKPSELDQGKYDADDNVKIICLGDSAVGKSKLMERFLMDGFQPQQLSTYALTLYKHTATV-------------DG----KTILVDFWDTAGQERFQSMHASYYHKAHACIMVFDIQRKVTYRNLSTWYTELREFRP-EIPCIVVANKIDADINVTQKSFNFAKK---------------------------FSL-PLYFVSAADGTNVVKLFNDAIRLAVSYKQNSQ-------------------------DFMDEIFQEL-ENF----S-----LEQEEE---------DVP--------DQEQSSSIET-PSEEVASPHS------------------------------------------------------------------------
HsaRABL2B -MAED------------------------------KTKPSELDQGKYDADDNVKIICLGDSAVGKSKLMERFLMDGFQPQQLSTYALTLYKHTATV-------------DG----RTILVDFWDTAGQERFQSMHASYYHKAHACIMVFDVQRKVTYRNLSTWYTELREFRP-EIPCIVVANKIDADINVTQKSFNFAKK---------------------------FSL-PLYFVSAADGTNVVKLFNDAIRLAVSYKQNSQ-------------------------DFMDEIFQEL-ENF----S-----LEQEEE---------DVP--------DQEQSSSIET-PSEEAASPHS------------------------------------------------------------------------
MmuRABL2  -MAGD------------------------------RNRHCELEQEKYDTHENVKIICLGDSAVGKSKLMERFLMDGFQPQQLSTYALTLYKHTATV-------------DG----KTILVDFWDTAGQERFQSMHASYYHKAHACIMVFDVQRKITYKNLGTWYAELREFRP-EIPCILVANKIDADIQMTQKNFSFAKK---------------------------FSL-PLYFVSAADGTNVVKLFNDAIRLAVAYKESSQ-------------------------DFMDEVLQEL-ENF----K-----LEQKEE---------DTS--------GQEQSDTTKS-PSPS------------------------------------------------------------------------------
GgaRABL2  -MAEA------------------------------AEQPRDEAPLEAGAEEAVKIICLGDSAVGKSKLLERFLLDGFRPQQLSTFALTLYQHRARV-------------DG----KAVLVDFWDTAGQERFQSMHASYYHKAHACIMVFDVQRKVTYKNLNNWYKELREFRP-EIPCIVVANKIDADMKVTQKSFNFARK---------------------------FSL-PFYFVSAADGTNVVKLFNDAIKLAVTYKQNSG-------------------------DFMDEVMQEL-ESF----D-----LEKKSE---------NLS--------DQEESYPEEK-PPSS------------------------------------------------------------------------------
TruRABL2  -MACD------------------------------VSSAPELDQNDYDADEQVKIICLGDSAVGKSKLMERFLMDKYRPHQLSTYALTLYKHTATV-------------GN----KTVAVDFWDTAGQERFQSMHPSYYHKAHACIMVFDVQRKITYKNLANWYKELREYRP-EIPCCVVANKIDVDLKVTQRSFNFGKK---------------------------QAL-PFYFVSAADGTNVVKMFREMIARAVDYKQNPS-------------------------DFMDEVLQEL-ENF----D-----LEKKEE---------NSE--------TDEDGLKAES-PELG------------------------------------------------------------------------------
PmrRABL2  -M-------------------------------------AAMEADECEADERVKIICLGDSAVGKSKLVERFLIDGYQPQQLSTYALTLYKYKATV-------------DD----KTVLVDIWDTAGQERFNSMHPSYYHKAHACVMVFDVQRKITYKNLASWYKELREYRP-EIPCVIVANKIDADYKVTQKNFNFAKK---------------------------QGL-PFYFVSAADGTNVVKLFRDAIKLAVSYKQNSK-------------------------DFMDEVMREL-ENF----E-----LESKGV---------SSE--------EDDNSKSK-------------------------------------------------------------------------------------
CinRABL2  -M-------------------------------------SSVIKNKSEDVEKLKIICLGDSAVGKSKLVERFLVDGYKPQQQSTYALTLFNYETKL-------------NG----KKISVDFWDTAGQERFSSMHPSYYHQAQACILVFDATRKVTYKNLNNWYKELRQYRP-NIPCLLVANKIDVDLGVTKRTFAFGTK---------------------------HNL-PQYFVSAANGTNVVKVFRDAIKAALSYKQNPT-------------------------DFVDQVLQEL-EHL----G-----NTEEDLI----HITCKTEE----------------------------------------------------------------------------------------------------
BflRABL2  -MADL------------------------------RASVPEYDK--EDGEYKVKVICLGDSAVGKSKLVERFLMDGYQPQQLSTYALTLFRYQTEV-------------GG----KDVCVDFWDTAGQERFNNMHPSYYYQAHCCILVFDVTRKVTYKNLPNWFKELRQYRP-EIPCLVCANKIDVDYKVTQKSFNFAKK---------------------------HQL-PFYFVSASDGTNVVKMFREAIQSAVGYKENTT-------------------------DFTDLVMREL-ENL----D----DLSLSNDV--------DSG--------VDTAGNSAET-HTDEADEEQAEGATGTT-----------------------------------------------------------------
SpuRABL2  -MANH------------------------------TNSSSVPEGDGKIAENGVKIICLGDSAVGKSKLVERFLMDGYQPQQLSTFALTLFRYETKV-------------DG----KEIVVDFWDTAGQERFSSMHPSYYHQAHACILVFDVTRKVTYKNLSQWYKELREYRP-EIPCVVAANKIDVDYKITQKNFNFSKK---------------------------HDL-PFFFVSAADGANVVKMFREAIKQAVSYKENPT-------------------------DFTDEIMKEL-EEM----D-----LKLSP----------DSG--------LESATNSEGK-ENGNVNDGATTSS---------------------------------------------------------------------
SkoRABL2  -MA--------------------------------ETAPMDPENAKIDRENSVKIICLGDSAVGKSKLVERFLMDGYQPQQLSTYALTLFRYNTTV-------------NS----KEVVVDFWDTAGQERFSSMHPSYYHQAHCCILVFDITRKITYKNLPNWFKELREYRP-SIPCICAANKIDVDYNVTKKAFNFAKK---------------------------HNM-PFYFVSASDGTNVVKLFRNAIKLATEYKENST-------------------------DFMDEVMREL-DNF----D----ELNLSS----------DSG--------LDTAPNSEEK-DEKEA-----------------------------------------------------------------------------
PhuRABL2  -MAENQ-----------------------------KISDNVDEKNIKKRKSYVKIICLGDSAVGKSKLVERFLLDNYKPQQISTYAVQLYRYETTV-------------NK----ETISVEFWDTAGQEMFQSIHPSYYHEANSCILVFDVTRKITYKNLPKWLKELRVFRP-NIPVLCAANKIDANMESTQMSYAFPQK---------------------------NNF-PFYYVSASNGTNVVCLFKDAIEAAVNSKKDSK-------------------------DITDQILEEL-ERL---------------------------------------------------------------------------------------------------------------------------------
FocRABL2  -MD--------------------------------GDATQDYDGKSENGAEVVKVICLGDSAVGKSKLLERFLLDKFKPQTISTYALTLYRYSSKV-------------KE----NDVIVDFWDTAGQEMFQSMHPSYYHQAHACIMVFDATRKITYKNLERWYKELRQYRP-HIPVFCAVNKIDANMDVTQQRFAFPEK---------------------------NSI-PLYYVSASNGMNVVKLFRDAIEAAMVYKKDPP-------------------------DVTDQIFQEL-QRL---------------------------------------------------------------------------------------------------------------------------------
ZneRABL2  -MAGR------------------------------EDDEIDYDDKERKGDLAVKVICLGDSAVGKSKLVERFLLDGYKPQQHSTYALTLFRYPTKI-------------EN----ESIIVDFWDTAGQEMFQSMHPSYYHQAHACILVFDATRKVTYKNLATWYQELRQYRP-HIPVFCAANKIDANMDVTQKAFAFPQK---------------------------HNI-PLYYVSASDGTNVVKLFRDAIQSAVKYKKNPS-------------------------DVTDQIIEEL-ERI---------------------------------------------------------------------------------------------------------------------------------
DpuRABL2  -MCD-------------------------------SNIGIDYEATGKSNDLAVKIILLGDSAVGKSKLIERFLLDDYKPHQLSTYALTLFRYKTQI-------------QE----KSVSVDFWDTAGQERFESLHPSYYHQANCAILIFDTVRKITYKNLKKWYDELRQYRP-EIPCFCAANKIDENFEATHKSFAFPTK---------------------------HRL-PFYYVSASDGTNVVKLFNDAINAAVTYRINPT-------------------------DLSDQIMDEL-EAL----K----------------------------------------------------------------------------------------------------------------------------
SmtRABL2  -----------------------------------MEEQPDYDLKGKSGELRVKVICLGDSAVGKSKLVERFLIDEYKTQQLSTYALTLFQYKTKI-------------ND----DEVVVDFWDTAGQERFNSMHASYYHQAHACILVFDTTRKITYKNLSTWYNELREHRP-EIPCFCVANKIDVDYKVTQKAFNFPKK---------------------------QNL-PFYFVSASDGTNVVKLFKDSIKAAIKYKKNPS-------------------------DFTDEILEEL-EHM----N-----TGNGV----------DFE--------DDESVSS--------------------------------------------------------------------------------------
MocRABL2  -MKGQ------------------------------FDDVSDYDQVGSDGVHRVKIICLGDSAVGKSKLVERFLLDEYKSNQLSTYALTLFRHKATV-------------DG----VNVAVDFWDTAGQERFQKLHASYYHQAHACVLVFDVTRKITYKNLQKWYEELRLQRP-TIPCICVANKIDADYDVTKKSFSFPLK---------------------------HNM-PIFYVSASDGTNVVKLFNEAIRAALQYKKNPT-------------------------DFVDNILEEL-QGV----E-----PRETCD---------GMKHH-----VVDDSRPISAT-----------------------------------------------------------------------------------
LheRABL2  MMTDEHSFPDG---------------------RPRSPGEVDYNSIEEDGSLRLKIICLGDSAVGKSKLVERFLLDGYKPHSLSTYALTMFQYKTKV-------------DD----ELVLVDIWDTAGQEKFNNLHPSYYHDAHACIMVFDSTRKPTYKSLTRWHTELREYRP-EIPCLCAANKIDANPSVTKKSFNLPQK---------------------------LKM-PLYFVSASDGTNVVKLFNDAIRSAVMYKKNPT-------------------------DFFDKVMQEL-EAV----N-----ISGDSY---------DASG------SFDANDFSEMS-GQGDCSNK--------------------------------------------------------------------------
EroRABL2  -MADSKKDS--------------------------DWESYDYDKEVKPGQTKIKVICLGDSAVGKSKLIERFLMDGYRPHQLSTYALTLFRYETKI-------------ND----AEVVVDFWDTAGQERFNNMHPSYYHQAHSCILVFDVTRKVTYKNLPEWYKELREYRP-EIPCLCAANKIDVENKITQKNFNFAKK---------------------------HNM-PLYYVSASDGTNVVKLFQDSIKAAISYKNNST-------------------------DFMDEIMKEL-ENF----D----NLELND----------SSP--------DSPRSENIPN-KTEDKKTVVVS-----------------------------------------------------------------------
PcaRABL2  -MSMA------------------------------DGKSPDYDQESKSGDTTVKVICLGDSAVGKSKLVERFLMDNYQPQQLSTYALTLFRYHTKL-------------NE----DNVCVDFWDTAGQERFSNMHPSYYHQAHACILVFDVSRKVTYKNLPMWLKELRDYRP-EIPCLCAANKIDVDYKITQKSFNFAKK---------------------------QGM-PFYFVSASDGTNVVKLFRDAINAAVSYKNNST-------------------------DYMDEVMREL-ETF----N----SMGLT-----------DSG--------FDTPDGAENK-SITDGPSIS-------------------------------------------------------------------------
LgiRABL2  -MA--------------------------------TTSMPDYDKTDGTDDVKVKIICLGDSAVGKSKLVERFLMDGYKPQQLSTYALTLFNYKTEH-------------NK----QSISVDFWDTAGQERFNNMHPSYYHQAHACIIVFDATRKVTYKNLPDWLKELREYRP-EIPCLCAANKIDADPGVTKRAFGFAKK---------------------------HNM-PFYFVSASEGTNVVKLFKDAIKAAVAYKNNPT-------------------------DFMDEIMNEL-ENF----E-----LETDKNIGEK-EEISDSG--------LDSPHSTDDK-PNS-------------------------------------------------------------------------------
CgiRABL2  -MANVE---------------------------------PDYEQKGEHGETKVKIICLGDSAVGKSKLVERFLMDGYKPQQLSTYALTLFNYQTEQ-------------GD----EKVSVDFWDTAGQERFNNMHPSYYHNAHACILVFDTTRKVTYKNLPNWLKELREYRP-EIPCLCCGNKIDVDYSITKKAFTFPKK---------------------------HGM-PFYFVSASDGTNVVKLFKDAIRAAVAYKNNST-------------------------DFMDEIMREL-ENF----E-----LETDNT-------TTDSA--------INSASNSGDK-KETDSS----------------------------------------------------------------------------
CteRABL2  -MA--------------------------------DASLPDYDKS-DGTCDKVKIICLGDSAVGKSKLVERFLMDGYQPQQLSTYALTLFKYKTTL-------------GD----QDVHVDFWDTAGQERFNNMHPSYYHQAHACIIVFDITRKVTYKNLPNWYKELRDYRP-EIPCLCAANKIDVDDSVTKKSFNFPKK---------------------------HKM-PFYYVSASDGTNVVKCFRDAIRAAVAYKNNST-------------------------DFMDEIMKEL-ESI----D-----LEPENG---------EATNIGK-------------------------------------------------------------------------------------------------
HroRABL2  -----------------------------------MSDIPNYDYLSADGLEKVKIICLGDSAVGKSKLIERFLLDGFKPQQLSTYALTLFKHRARI-------------DD----KDVLVDFWDTAGQERFSSMHPSYYHQAHACVLVFDVTRKVTYKNLPNWYKELREHRP-EVPCLCVANKIDVDMDVVKKSFNFATK---------------------------NKL-PLYFVSASDGSNVVKVFKDAIRAAVAYKNNST-------------------------DITDEIMKEL-EIY----N-----MEVGNA---------NDS--------NNNNESNDSN-----------------------------------------------------------------------------------
CsiRABL2  -----------------------------------MSEVPDYD--QDEFENKVKIICLGDSAVGKSKLVERFLMNDFHPQQLSTYAVTLYKHKAEV-------------DN----RRISVDFWDTAGQERFQSMHASYYYQAHACIFVFDVTRKITYKNLPKWYEELRQYRP-DIPCLCIANKIDADMTMTNKTFNFAKK---------------------------NKM-ALYFVSASNGTNVVRAFRDAIRAAVAYKESAT-------------------------DIMDQIMEEL-ERM----Q-----LDNCNE---------AAG--------DLEHTTSNAT-D---------------------------------------------------------------------------------
FheRABL2  -M-------------------------------------ADSPSFRATSETVVKVICLGDSAVGKSKLLERFLIQEYHPQRLSTYAVNLYHHETQI-------------DG----KKIQVDFWDTAGQERFQNIHASYYHQAHACIFVFDVTRKVTYKNLGRWYEELRQYRP-DIPCLCLANKIDVDSSVTNKTFKFPSE---------------------------NKM-ALYFVSASDGTNVVRAFDDAIRAALAYKETTC-------------------------DIMDHIMEEL-KNM----G----DGGVPGR---------RNN----------------------------------------------------------V------------------------------------------
EgnRABL2  -----------------------------------MSEISDRDAIVIGKSCRVKVICLGDSAVGKSKLVERFLLSGYQSNQLSTYALNIYKHQMTI-------------ED----RLIDVDFWDTAGQERFQSMHPSYYHQAHACILVFDVTRKITYKNLNNWLSELRKYRP-EIPCFCAANKIDTDTEVTNKAFNFAKK---------------------------NSI-PFYFVSAANGTNVVRVFTDAVRAAVAYKEDST-------------------------DPLDQIMEEL-EVM----E-----QNHEVN---------DGES------SYADPLS---------------------------------------------------------------------------------------
TsoRABL2  -----------------------------------MSEISDQGAVDVRKSCRVKVICLGDSAVGKSKLVERFLLSGYQSNQLSTYALNIHKHQMTL-------------ED----RLIDVDFWDTAGQERFQSMHPSYYHQAHACILVFDVTRKITYKNLSNWLLELRKYRP-EIPCFCAANKIDTDTEVTNKAFNFAKK---------------------------NNM-PFYFVSAANGTNVVRVFTDAIRAAVAYKENSA-------------------------DPLDQIMEEL-EDM----E-----RNHEVN---------DGD-------SYADPLP---------------------------------------------------------------------------------------
SmeRABL2  -----------------------------------MAEPPDYDIKDPNCDVRVKIICLGDSAVGKSKLVERFLMDGYQPQQLSTYALTLFKYQTNL-------------DG----KKLTVDFWDTAGQERYQSMHTSYYYQAHACILVFDVTRKITYKNLDNWYKELREHRP-DIPCICVANKIDADMSATKRSFKFPTK---------------------------NKM-PFYFVSASDGTNVVKAFRDSIKLAVGYKENAT-------------------------DIMDQILEEL-DNM----E-----LETDDN---------FFN-----------------------------------------------------------------------------------------------------
MliRABL2  -----------------------------------MSEIPDYESSHEGYDSRVKIICLGDSAVGKSKLVERFVMNGYQPQQLSTYALTLFKHEYQL-------------NG----KKIAIDFWDTAGQERFQSMHTSYYHQAHCCILVFDVTRKVTYKNLPTWYKELRQHRP-EIPCLCAANKIDANMAITKTSFNFPKK---------------------------HGM-PFYFVSASDGTNVVKMFRHAIQAAVEYRTSGS-------------------------DITDLIMEEL-ERM----E-----LEETEQ---------TSP-----------------------------------------------------------------------------------------------------
AvaRABL2A -MS---------------------------------------ESVPLSTSDSLKIIVLGDSAVGKSKLLERFLVKNFEDSRYSTYAVNIFKHTTKI-------------DN----KPVEVEFWDTAGQEKFDNLHYSYFHQAHACIMIFDATRKITYKNLDRWYTELRAIRP-HIPCVCAVNKIDAAMEITKKSFSFPKK---------------------------HDM-PLYFVSAANGTNVVRLFRDVIRLAQAYRAGDS-P-----------------------DFIDQIMREL-ETM----N-----DDNNAN---------TPSND---------------------------------------------------------------------------------------------------
AvaRABL2B -MS---------------------------------------ESVPLSTSDSLKIIVLGDSAVGKSKLLERFLVKNFEDSRYSTYAVNIFKHTTKI-------------DN----KPVEVEFWDTAGQEKFDNLHYSYFHQAHACIMIFDATRKITYKNLDRWYTELRAIRP-HIPCVCAVNKIDAAMEITKKSFSFPKK---------------------------HDM-PLYFVSAANGTNVVRLFRDVIRLAQAYRAGDS-P-----------------------DFIDQIMREL-ETM----N-----DDNNTN---------TPSND---------------------------------------------------------------------------------------------------
NveRABL2  -MAARENE---------------------------VDSFDDLTSSKKDKETSVKVICLGDSAVGKSKLVERFLMDGYKPQQLSTYALTLFHYETKV-------------DD----KDIRVDFWDTAGQERFSSMHPSYYHQAHACLLVFDITRKITYKNLANWYKELRKYRD-SIPCIVVANKIDVDYKVTQKSFNFPKK---------------------------YGL-PFYFVSASDGTNVVKVFREAIRLGVNYKENST-------------------------DFMDMVMQEL-ENF----D----ELSTTQ----------ESE--------FDNKSDEENS-----------------------------------------------------------------------------------
HvuRABL2  ----------------------------------------------MDTEEKVKVICLGDSAVGKSKLVERFLMDGYKPQQLSTYALTLFKYFTKI-------------DD----RNVHVEFWDTAGQERFSSMHPSYYHASHACILVFDVTRKTTYKNLSNWYKELRAYRP-DIPCILVANKIDVDYNSTKRSFNFGKK---------------------------YHL-PFYFASASDGTNVVKVFREAIKAGLHYKDNST-------------------------DFVDEVLKEL-ENF----D----DMSLQN----------PANGLSN---GSDLTTSNVAS-SSKDK-----------------------------------------------------------------------------
TadRABL2  -MAASEE----------------------------IHEDFDYDNDDDSTTAKIKIICLGDSAVGKSKLVERFLMDDYKPQQLSTYALTLFRYNTTM-------------NG----KSIGVDIWDTAGQERFASLHPSYYHQAHACILTFDVTRKITYKNLSRWYKELREYRP-SIPCVVVANKIDVDFSITKKSFNFVKK---------------------------HNL-PFYFVSASDGTNVVKVFRNAIKLAVVYKEEST-------------------------DFLDEVMREL-ENL----D----DLSLSE----------NQP-------KLGEYDDNVES-----------------------------------------------------------------------------------
AquRABL2  -----------------------------------MAAISSMDSSDPSSQSDIKIICLGDSAVGKSKLVERYLMDKFKPQRHSTYAVTLFAHKAEI-------------ND----RTISVDLWDTAGQERFQSMHPSYYHGAHGCILVFDVTRKITYKNLAHWYSELRESRP-KIPCILLANKIDVDMKVTKTSFKFASK---------------------------HKL-PFYFVSARDGTNVVKAFRDITRAALAYKETSQ-------------------------DLVDQILEEL-REM----Q-----NEGMSD---------EES--------DSHTTPDSNH-DLEKVT----------------------------------------------------------------------------
OcaRABL2  -MATS----------------------------------RAVEEVEDGDVYKVKIICLGDSAVGKSKLVERFLMNGYQPRQQSTYALTRFKYSTKF-------------KG----KPVEVDIWDTAGQEKFHTMHAVYYHQAHACVMVFDVTRKVTYKNLGIWYKELRNYRP-EIPCILVANKIDADMSMTQKSFNFPKK---------------------------YKL-PFYFVSASDGSNVVKVFKEAIKAAIIYKESST-------------------------DFVDEVMREL-EAL----D-----GLKGGQ---------PAK--------SDSSLSGSQG-DPFDSPEDATH-----------------------------------------------------------------------
MleRABL2  ----------------------------------------------MAAKEKVKVICLGDSAVGKSKLVERYLKDNYKPQQLSTYALTLFKHVDTV-------------DD----RQVELELWDTAGQERFQSMHSAYYHGAQACILVFDVSRKITYKNLTSWYKELRKYRP-KIPVICVANKIDKDPDMAGKSFKFATA---------------------------NNI-EMFFASAADGSNVVKIFREAIRLALHYKENTE-------------------------EYEDHVLEEL-RLH----S-----PDENDF---------DYEPE-----AEDS------------------------------------------------------------------------------------------
PbaRABL2  ------------------------------------------------MTEKVKVICLGDSAVGKSKLVERYLLDNYKPQQLSTYALTLFKHKDIV-------------DD----QEVELELWDTAGQERFQSMHSAYYHGAQACILVFDVSRKVTYKNLAIWYKELRKHRP-KIPVICVGNKVDTDLSMANKAYKFPQS---------------------------NNI-PLFFSSAADGSNVVKIFREAIRQAVHYKNNTE-------------------------EYEDHVLEEL-EYL----N-----AFDDDA---------DD------------------------------------------------------------------------------------------------------
MbrRABL2  -M--------------------------------------------PTAENGVKVICLGDSAVGKSKLVERFLMDDYKPHQLSTFALTLFRHNAKV-------------EG----KDVVVDFWDTAGQERFASVHPSYYHEAHSCILVFDVTRKVTYKNLLTWYKELREYRP-KIPVIVVANKIDVDYSVTNKAFGFPKK---------------------------HNL-PFYFASASDGTNVVKIFNEAIANGVRYKANSD-------------------------DFVDQVLELLHED---GFD----------------------------------------------------------------------------------------------------------------------------
SroRABL2  -MADDV---------------------------------AKLAETSGTGENTVKVICLGDSAVGKSKLVERFLMDDYKPHQLSTYALTLFKHTATV-------------GD----KEVVVDFWDTAGQERFSSMHPSYYHEAHSCILVFDITRKVTYKNLQTWYKELRKYRP-KIPVICVANKIDIDYSVTQKSFGFPKK---------------------------HGL-PFHFVSASDGTNVVKVFNEAIESAVQYKEHSD-------------------------DFVDQVMQLLHED-----K-----FAGDDDDG-------DAN-----------------------------------------------------------------------------------------------------
MvbRABL2  -MKH----------F---------FC---KSRMEPSAPAGSSNPADSEDVLSVKIICVGDSAVGKSKMLERFLMDDYQPHQLSTYALTLFQHTTTV-------------DG----EKVRVDFWDTAGQERFSSMHPSYYHQAHACILAFDCTRKITYKNLSTWFSEMRGYRP-HIPCICVANKIDVDYSVTQKQFQFAKK---------------------------HDM-PFYFVSASDGTNIVKVFTNAIKMAVEYKKQSP-------------------------DFVDQVMELLSTT-----P-----DAAVNK---------EVE--------DDEN-----------------------------------------------------------------------------------------
OboRABL2  -MFKASTESLS------------------AAEAPREPVSPEYDPGPGVKQADLKIILLGDSAVGKSKLVERYLLNSYVHQHTSTYALTLYRHNVPHPDEARMR------NG----ETLSIDFWDTAGQERFQSMHPSYYHAAHACLLIFDITRKITYKHLAAWHSQLTSHRP-NIPIILVANKIDLNLQATKSHFAFSDT---------------------------PGIVSTHFVSASDGTNVVKAFREAIGRAYEYKAEGA-K-------------------GAGKDFVDEVIGLLEDD-----SLFGFDAPDGETSAP------KAGKEVAAAAPDEKKPRPAANAEVAKGGAAH-------------------------------------------------------------------------
AmaRABL2  -MPV------------------------------------------EDQPADLKIILLGDSAAGKTKLVERFLVQSYSQQTSSTYAITVFRHSTKHPT-----------TG----QSLAIDFYDTAGQERFASMHASYYYGAHACLLCFDLTRKVTYKNLDNWYRELRQYRP-KIPVLILANKVDVQPEMAKRDFKFAVE---------------------------NQL-PVMFVSASDGTNVVAAFETIISRALDYKASPD-R-----------------------DFMDDVLDMLRDT-----P-----SPATALAPL--------------------------------------------------------------------------------------------------------------
CanRABL2  -M-------------------------------------PEPTSTLAERPADLKIILLGDSAVGKSKLIERFLLKDYSQATSSTYALSLFKYKSTHPT-----------TG----EPLHIDFYDTAGQERFASMHPGYYHGAHACLLCFDLSRKITYKNLDNWYRELRQYRP-NIPVIVLANKIDQQPEMAKRDFKFAVD---------------------------NRL-DLVFVSASDGTNVVMAFQDIIRRAIAYKESDT-R-----------------------DFMDDVLDLIREG-----S-----TPASEATAI----------------AAPAAAAPSSSTMPVHIP----------------------------------------------------------------------------
BdeRABL2  -MSTKP-------------------------------------LGSVSQPADIKIILLGDSAVGKSKLMERFLLDDYVPHQQSTYALTLYRHNAIHPKSTSN-------PP----EKIAVDFWDTAGQERFQSMHASYYMGAHCCILVFDVTRKITYKNLDTWYEELVQHRGIKMPVIVVANKIDMDPSRGSKSFGFVEKRRQERG------------------GSVDDL-PFYFVSASDGSNVVAIFLEAIQRGFEYKNKMV-I-------------------DKQGTFVDDVLQFIQEE-----E-----NRPDGLF---------AKQI-----LVDSNT----------------------------------------------------------------------------------------
SpnRABL2  -MS------------------------------------ATQAKNASEQPADLKIILLGDSAVGKSKLAERFLLDEFHPQQLSTYALTLYRHVCTYPFPSDRRSGASTEKP----RKLRVDFWDTAGQERFQSMHAGYYHGAHACILVFDVGRKITYKNLDTWYDELVSHVGLKLPVIVVANKIDVDPSRAKRAFGFVERRRAERAEASGTAPQNNDPEEPQDPSQSDHL-PLFFASASDGTNVVSIFREAINRAVKFKEQGE-G-----------------------TFVDEVLAFIREE-----E-----GKEGGLFA-----------------REDEGITPSITAH---------------------------------------------------------------------------------
GprRABL2  ---------------------------------------MGDRSQSAERPADVKIILLGDSAVGKSKLIERFLLSNYSPQQHSTYALTLYRHTVPHPAD----------SS----RQLTVEWWDTAGQERFQALHPSYYHRAHACLLVFDLTRKVTYKNLEAWYSELTKYRSDKIPVVVVANKVDAEPDRAQKSFAFVDRRKRCRD---------------------EDL-PLFCVSASDGTNVVAAFQEAIRRAVNFKESSS-SISCLPRTSSSQPRSTVQPESNQADFVDEVLRFIEEE-----E-----QRPDGIFRGA-----GTKDEDW--DKEDASRAGMAV-------------------PVAKGWREG-----------SVGAG---------------------------------------
PirRABL2  -MATTATDF------------------------------SGIKNDANHKPCDLKIILLGDSAVGKSKLIERFLLNDYIPYQLSTYALTLYRHTCPHPLNP---------KA----PPISVEFWDTAGQERFQSMHPSYYHMAHVCILCFDLTRKITYKNLQHWYDELMKYRN-NIPVVVIANKVDAEPERAKRRYGFVERKKKGEA---------------------EDL-PLYFVSASDGTNVVSAFNDAIKRGMKYKESEK-N----------------------KDFVEEVLNFIAEE-----E-----KRPDGIF---------SKKEDT-DIFMDNKTTQLANA----------------------------------------------------------------------------------
PpoRABL2  ---------------------------------------------MSERADAVKIILLGDSAVGKSKLVERFMMNAYQPQQLSTYALTLFQHDADI-------------DG----QKVKVDFWDTAGQEKFNSIHPSYYYRAHACILAFDITRKVTYQNLTNWYNELQENRK-GIPIIVVANKIDVDYSVTSKAFTFASK---------------------------RDL-PFYFVSSSDGTNVVKVFTEAIKLGMKYKANPP-Q-----------------------DFLDEVLDLLENE-----S-----PLKKTAST---------------------------------------------------------------------------------------------------------------
MbaRABL2  -----------------------------------------------MTDKPIKVILLGDSAVGKSKMMERFLREEYNPDNSSTFGVTIHRHATEV-------------GD----KDVMIEFWDTAGQPTFKELHPSFYDRAWGCVLVFDITRKETYTHLNEWYTELLQYRK-GIPCCLVANKIDVDPTVEGRSFNFATK---------------------------HKL-PLYYVSAASGKNISQPFDDIVKRAYECLLNPP-D-----------------------DIIDDVLDLIQ------------------------------------------------------------------------------------------------------------------------------------
McaRABL2  -MSRRPS----------------------------TAEETSDSAEIRARSDRVKVILLGDSAVGKSKLVERFLMDAYHPQQLSTYALTLFEYTAEI-------------DG----QSVHVDFWDTAGQERFNSMHPSYYHRAHVCILVFDVTRKLTYTNLENWYRELQEYRK-GIPVIVVANKIDYDYRVTQKTFAFATK---------------------------HNL-PFYFASASDGTNIVKLFNDAIRLGLTYKESPSEE-----------------------DFVALVEKILDES-----P-----LNRPSSA--------PAK-----------------------------------------------------------------------------------------------------
MalRABL2  -MAGK------------------------------QSDAGGDPRELEARADNIKVILLGDSAVGKSKLVERFMMDAYHPQQLSTYALTLFEHNAEI-------------DG----RKVKVDFWDTAGQERFNSMHPSYYYKAHVCLLVFDVTRKVTYKNLSNWYKELQEYRK-GIPVIVVANKIDVDYRVTQKTFNFAVK---------------------------HGM-PFFFASASDGTNVVKMFQEAIRMGLQYKENPP-DA--------------------ATDFIGSVMSLLDEG-----P-----LIKK-------------------------------------------------------------------------------------------------------------------
TpyRABL2  -------------------------------------------MDDVPERNKLKIILLGDSAVGKSKLIERFLLDKYRPQQSSTYALSWYRYEATV-------------GT----EKILIDFWDTAGQDTFNSVHPSYYQDANACILCFDVTRKITYQNMKQWYAELQEHRA-GIPVCVAANKIDMDMKAAKKAYKFPEK---------------------------HHL-PVYFVSASNGINVVKLFQEAIQMAFGAKDTAG-D-----------------------ESYNNIMRFLREEFH--GD----ELEDDLDT--------STPSAT-----PPAALRPGPPPPPASATQ-----------------------------VVSATVA-------PPPPPGRPRRM---------------------
MonRABL2  -MSD-----------------------------------ESNDEHISASGSAIKVIILGDSAVGKTKLMERFLLDKFHKQQLSTYALTIFPYTAHLQ------------DV----GEVKVEFWDTAGQDIFEEIHQSYYYGAHSCILCFDVTRKITYTNMQKWWDELQKYSP-NIPSVVVANKIDVDPSATTKEFAFAKK---------------------------HGL-PLYYCSASTGVNVVKLFTDAINLGYRTMKNPT-D-----------------------PFMGELMRLLEEN-----R-----SANPDLLPRPA----PSPQTY-----SISMSPSFPSTPTPTNSIDSADSSTFTSSSSTPSESSTFSSEFQKTSEAQKHPITASISQSTRSSLPSSASPDNTSTVRQAPPPPPPPPKKKY
NfoRABL2  -M--------------------------------------------SNSSKVVKVILLGDSAVGKSKLVERFLIDGYREQQQSTFALNIFPYRAKI-------------DG----EDCDVEFWDTAGQERFNNVHPSYYHQAHACILVFD-DRQITYKNLEKWYNELQTYRK-GIPAIVVCNKIDLNMDVTKTTFNFATK---------------------------REL-PLYYVSAADGTNVVKAFTDALKLAKKCRDNPE-K-----------------------EFVDDVLELLKD-----------------------------------------------------------------------------------------------------------------------------------
NgrRABL2  -----------------------------------------------MSNKVIKVILLGDSAVGKSKLVERFMIDGFREQQQSTFAVNIFPYKAKI-------------DG----EDCDVEFWDTAGQERFNNVHPSYYDRAHACIFVFD-DRQITYKNLEKWYNELQTYRK-GIPSIVICNKIDLNMEATKTTFNFASK---------------------------REL-PLYYASAADGTNVVKAFTDALKLAKKCRDNPE-K-----------------------EFVDDVLELLN------------------------------------------------------------------------------------------------------------------------------------
RamRABL2  -MASSSRAPPPPARSSSA---------AAAAAAAPAVGDDEGDELNESHKDEIKIILLGDSAVGKSKLVERFLMDNYQPQRQSTYALTLFQYEADI-------------DG----EKVPVGIWDTAGQERFNSLHPSYYHRANACILVFDTTRKVTYKNLDRWYAELQEYRK-AIPCLVVANKIDVDYSVTNKSFNFATK---------------------------RNL-PFYFCSASDGTNVVKLFHDAIRMGFEAKKNPQ-K-----------------------DFYDAVLDLLS------------------------------------------------------------------------------------------------------------------------------------
MpnRABL2  -MAE----WRVTTD--ASETDTGAFLKALREQEGRSGDVEDAGPSLVTSKGAVKVILLGDSAVGKSKLVERFLMDGYQPQQLSTFALTLFRYEFKHP------------DG----RLVAVDFWDTAGQERFNNLHPSYFYKAHACVLAFDVTRKVTYKNLDRWYHELREYCP-GIPTICVANKIDIDYRVTERSFAFPTK---------------------------HKL-PFFFVSASDGTNVVKVFQQAIMMGLDHKENPS-D-----------------------DFMTMALELLGE-----------------------------------------------------------------------------------------------------------------------------------
MpcRABL2  -MADDGHFPRVTTDDTAAATDTEAFLKALKVQEGKTGEEGKDELSLVTSKGAIKIILLGDSAVGKSKLVERFLMDGYQPQQLSTFALTLFRYNFNHP------------DG----RVVAVDFWDTAGQERFNNLHPSYFYRAHACILAFDVTRKVTYKNLDRWYRELEEYCP-GIPTIVVANKIDVDYRVTERTFAFPER---------------------------RNL-PFAFVSASDGTNVVKTFQQAIMMGIKQKENPS-D-----------------------DFLTMALELLAE-----------------------------------------------------------------------------------------------------------------------------------
AstRABL2  -M-QSPG----------------------------KIGPHNEGLTLLTGSNAIKLILLGDSASGKSKLVERVLLDEYKPHQLSTYALTLYRHNYKGD------------DG----VSTPVDIWDTAGQDRFNSLHPSYYYRAHVCLMVFDVTRKVTYKNLDRWYDELRQFCP-SIPVLVLANKIDLDMSVTARSFGFATK---------------------------RDL-PLFYVSASNGTNIVKAFQAAMAAGLQYKCAPK-D-----------------------DFYSEVVDLLGEL-----E----------------------------------------------------------------------------------------------------------------------------
CreRABL2  -MADGAS----------------------------TSGPQGEELTLLTGSDAIKIILLGDSAVGKSKLVERFLLDNYKPHQLSTYALTLYRYNFKTQ------------DA----KTVACDIWDTAGQERFNSMHSSYYYRAHACIMVFDVTRKVTYKNLEKWYDELQDNCK-GIPTLVVANKIDIDYKVTSKSFNFAAK---------------------------RKL-PFFFVSASDGTNVVKIFNMAIMAGMRWKAAPK-D-----------------------DFYQEVLDLLGEI-----S-----MDTRKQ--------------------LEDAVKALED-EKDKDGEGQ-------------------------------------------------------------------------
VcaRABL2  -MTDGAS----------------------------TSGPQGEELTLLTGSDAVKIILLGDSAVGKSKLVERFLLDNYKPHQLSTYALTLYRYNFKTQ------------DD----KVVPVDIWDTAGQERFNSMHSSYYYRAHACIMVFDVTRKVTYKNLEKWYDELQEHCK-GIPTVVVANKIDIDYKVTSKSFNFAAK---------------------------RKL-PFFFVSASDGTNVVKIFNMAIMAGMRWKAAPK-D-----------------------DFYQEVLDLLGEI-----S-----MDTRKQ--------------------LEDAVKALEE-GKEKDEGGN-------------------------------------------------------------------------
CpxRABL2  -MSEAE-----------------------------PVHQSPASPPDLSNNDVVKVILLGDSAVGKSKLVERFLMDGYKPQQLSTYALTLYRYVDDV-------------DG----KQVPVDFWDTAGQERFNSMHPSYYYRANACIMVFDVTRKVTYKNLETWYKELQDFCK-GIPTLVVANKIDLDTKVTQKTFNFATK---------------------------RNL-PLYFVSASDGTNVVKIFKEAVRLGIKYKEMPP-Q-----------------------DFYQEVLSLLSDS-----T-----LGNKASG--------EAGD------YGDEDKDSLRPKSSGTAGTGSRGPSP--------------------------RP----------------------------------------
GthRABL2  -M-----------------------------------------AEKNSDEDVIKVILLGDSAVGKSKLVERFLMDGYEPRQDSTYALTLYRHETTV-------------DG----QPVSVDFWDTAGQERFNSMHPSYYYGAHACIMCFDITRKVTYTNLSKWWEELQEYRE-GIPCIVVANKIDLDMKVTKKTFNFAAK---------------------------RNL-PFYFVSASEGTNVVKVFKEAISLGYHYRHDPNKS-----------------------DFFSEVMDLLAEDVKEKLD-----------------------------------------------------------------------------------------------P----------------------------
CryRABL2  --------------------------------------------MAAVDEGVLKIILLGDSAVGKSKLVERFLMDGYEPRQDSTYALTLYRHDATV-------------DG----KKVAVDFWDTAGQERFNNMHPSYYYGAHACIMCFDITRKVTYTNLSKWWQELQEYRK-GIPCIVVANKIDLDMKVTKKSFNFAAK---------------------------RNL-PFSFVSASEGTNVVKVFTEAIKLGHQYQLNPDSE-----------------------DFLAEIMDLLHED-----D-----PPASGAP----------------------------------------------------------------------------------------------------------------
GavRABL2  -MAAEGEEGGAG-----------------------ESAEAPKKKPAARRDDALKCILLGDSAVGKSKLVERFLMDNYEPHQDSTYALTLFRYNTYV-------------EG----KEVAVDFWDTAGQERFNSMHPSYYYGAHACIMVFDITRKTTYQNLTKWYKELQEYRK-GIPCIVVANKIDLNMAVTKKSFNFAAK---------------------------RNL-PFSFVSASEGTNVVKVFNDVIQMAQHYRENPT-D-----------------------DFFADVMELINED-----Q-----LGVVGGA-------GGAS-----------------------------------------------------------------------------------------------------
PicRABL2  -------------------------------------AGEQAAQAPASTRDAVKIILLGDSAVGKSKLVERFLMDGYQPQQLSTYALTLFPYEAEV-------------DG----RKVSIDFWDTAGQERFNSLHPSYYYQAHACILAFDVTRKLTYKNLSSWFKELQEHRK-SIPCIVVANKIDXDRKATTKSFNFATK---------------------------RNL-DFYYVSAADGTNVVK-----------------------------------------------------------------------------------------------------------------------------------------------------------------------------------------
EhuRABL2  -MAEAAP--------------------------------QLPDGLPAAEENVIKIILLGDSAVGKSKLVERFLMNEYQPRQLSTFALTLFRHVGVV-------------GG----KQYQVDFWDTAGQERFNSMHPAYYHMAHACIMCFDVTRKQTYKNLPAWYKELREYRP-GIPVICVANKIDVDAKVTQKEFAFPKK---------------------------HNM-TLFYCSASDGTNVVLAFNKAIELAVEYSASPP-E-----------------------DFVDQVMGLISKT-----S-----ISPAITT--------GAS-----------------------------------------------------------------------------------------------------
PatRABL2  -MAPSEEALYRA----------------PGPDGPAGSSSGPNGSLDALGENALKIILLGDSACGKSKLVERFLLNNYQSRQLSTYALTLFRHNATV-------------DG----QKVEIDFWDTAGQERFNSMHPAYYHLAHACILCFDVTRKQTYKNLPDWYRELREFRQ-GIPVVCIGNKIDVDPKVTQKEFGFPKK---------------------------HNL-EFFYCSASDGTNVVAAFEAAISKAIEYSKKPP-E-----------------------DFVDQVMQLLDDG-----K-----PENRWT---------GAEGE-----RLAS------------------------------------------------------------------------------------------
PavRABL2  -MAAPDE--------------------------------GMKAPPTADSEEPIKIILLGDSATGKSKLVERYLVDNYAQHQLSTYALTLFRKDVEL-------------DG----KTYHVDFWDTAGQERFQRLHPSYYHRAHACILCFDVTRKITYKNLVTWYKELREFRK-NIPVICVANKVDVDLAVTGKAFGFPEK---------------------------HSI-PLFMCSAADGTNVVQAFDLAMKMAIECSKRPP-D-----------------------DFYEDVMALLAEG-----S-----ISEPK------------------------------------------------------------------------------------------------------------------
PsoRABL2  -MAAKSD--------------------------------TVEVLEDSLGEPDVKVILLGDSAVGKSKLVERFMMNEYQPRQLSTFALTLFRKEVGL-------------DGENGKRTVKVDFWDTAGQERFSSMHPSYYYGATACILVFDVTRKVTYQNLANWYKELREYCE-NIPCFLVANKIDVDMNVTSKKFKFAET---------------------------HNL-EFSFVSAADGTNVVKVFKDAVEAAYKFKREGG-------------------------DFMSDVLDLLGED-----A------K---------------------------------------------------------------------------------------------------------------------
SdiRABL2  -MPTDDE--------------------------------SKALQEDGLGDADLKIILLGDSAVGKSKLVERFMMNEYQPRQLSTFALTLFRKEFPMD------------DG----STAKIDIWDTAGQERFSSMHPSYYFGASACILVFDVTRKTTYQHLADWYKELREYCE-NVPCFLAANKIDVDLEVTNKKFKFAET---------------------------HNL-GFHFVSAADGTNVVKIFEEAIHAGLKYKKEGG-------------------------DFMADVLDLLNEP-----P-----RK---------------------------------------------------------------------------------------------------------------------
AanRABL2  -MSKEEESKSRDGE-----------------GKDEGKDGEEKDGAPFDDEAEIKLILLGDSAVGKTKMVERFLMDEYNPQQLSTYALTLFRKVVDI-------------DD----RKVSVDIWDTAGQERFNKMHPAYYHRAHACILCFDVTRKATYQHLSDWYDELRESCE-NIPCILVANKIDIDYQVTRKNFKFASS---------------------------KGI-PFYFVSAADGTNVVKVFRAAIEAALDWKKNGE-------------------------DFMTEALRLVEGP----------------------------------------------------------------------------------------------------------------------------------
EsiRABL2  -MSE----------------------------RKTEGKDHMEDGDEDLEDPDLKVILLGDSAVGKSKLVERYLMDEYNPRQLSTFALTLFRKNAKLD------------DG----VEVKVDFWDTAGQERFSSMHPSYYYRAHACIMVFDVTRKATYKNLSDWYGELRQYSE-SIPCLLIANKIDVDYQVTKKSFKFAQQ---------------------------HDL-PFFFVSAADGTNVVKAFDEAVKAGMEFKKNGG-------------------------DFVDEALSLLEGK----------------------------------------------------------------------------------------------------------------------------------
TmiRABL2  -MADEGKLSEGKDE------------------GKTANEGDNNNNGVDLGDPDCKIILLGDSAVGKSKLVERYLMDEYNPRQLSTYALTLYRKTVVLE------------EG----NTYDIDLWDTAGQERFNSLHPSYYYGAHCCILVFDVTRKVTYQHLSDWYGELRQYCE-SIPCILVANKIDVDYKVTKKSFKFASQ---------------------------YEL-PFFFVSAADGTNVVKVFHEAVRLGINFKKNNP-------------------------DFMTEVIEFLK------------------------------------------------------------------------------------------------------------------------------------
TthRABL2  -MDN------------------------------KSKKQVKEYDAKEFSPCDMKVILLGDSAVGKSKLVERFLLDDYEERNMSTYALTMYRHVANL-------------DG----KQYKIDIWDTAGQEQFQTLHASYYFQANCCILVFDITRKITYLNLKKWYTEMREHCP-DIPCILIANKIDADRNVTNKQFKFATQ---------------------------HNL-PFYFVSAADGTNVVKIFQEALRLALDNKINPP-N-----------------------KDDKFFEDLINDK-----D-----LFDDLDD----------------------------------------------------------------------------------------------------------------
ImuRABL2  -MD--------------------------------KKQNIKEYDEKEFVPCDLKVILLGDSAVGKSKLVERFLLNDYEERNLSTYALTMYRHVANI-------------DG----KQYKIDLWDTAGQEQFQTLHSSYYFKANVCILVFDITRKITYINLKKWYQEMREYCP-DIPCILIANKIDVDRDVTNKQFKFASQ---------------------------NNL-PFYFVSAADGTNVVKVFHEALKLALDNKLNPP-D-----------------------DFMKEVDDLLNDN-----N-----IFGKKQ-----------------------------------------------------------------------------------------------------------------
PteRABL2A -M----------------------------------KRKVREYDENEFSKADLKIILLGDSAVGKSKLVERFLLDDYEERQQSTYALTMYRHNAKF-------------EG----KTYKIDLWDTAGQECFQTLHASYYYGAHACILCFDVTRKITYTNLKKWYEEMRQNCP-TIPCLLVANKIDLDPSVTETKFKFAES---------------------------NNL-PIYYTSSADGTNVVKVFQEALKAAIEHKQKPG-G-----------------------QFMDDLMDYLQG-----------------------------------------------------------------------------------------------------------------------------------
PteRABL2B -M----------------------------------KRKVREYDENEFSKADLKIILLGDSAVGKSKLVERFLLDDYEERQQSTYALTMYRHNAKF-------------EG----KTYKIDLWDTAGQECFQTLHASYYYGAHACILCFDVTRKITYTNLKKWYEEMRQNCP-TIPCLLVANKIDLDPSVTETKFKFAES---------------------------NNL-PIYYTSSADGTNVVKVFQEALKAAIEHKQKPG-G-----------------------QFMDDLMDYLQG-----------------------------------------------------------------------------------------------------------------------------------
OtrRABL2  -MDKQKT---------L------------------ENLDKKVQKAKEIPLADLKLILLGDSAVGKSKLTERFLLNDYEERTSSTHALTMYRHNTTV-------------NG----KEYKVDIWDTAGQESFNELHPSYYFGAHAAILVFDASRKVTYQNLKGWYKEMRNQCP-KIPCIIIANKIDIDERATQRKYKFVED---------------------------LGV-PFNFVSAADGTNVVLIFREALDMAIKYKEDPDND-----------------------DFMKEVMDLLGDDI---------------------------------------------------------------------------------------------------------------------------------
SmiRABL2  -MAEETPAP------------------------------PGPLAEDDHAPADLKIILCGDSAVGKSKMVERFLLEEYNPRSLSTFALTLFRYHHTAE------------DG----RRWTIDFWDTAGQEQFVKLHASYYFQANACILAFDVTRKITYKNLETWYQEIRHYCP-DIPVVCVANKIDVEPAMAKKKFNFPVT---------------------------HQL-PFFFVSCADGTNVVRVFKEAISLAIKNKEHPP-D-----------------------EVLAEIYALLAED-----G-----RPSKTD---------DAAEV-----AYDEAPAEAVPVDPPLPPAPPA------------------------------------------------------------------------
PmaRABL2  -MATSESQSYFD-----------------------QTEHRQEYAEDDMTPADLKIILLGDSASGKSKLVERFLLDDYNPRQLSTYAVNLFRYTTATE------------DG----RQWKVDLWDTAGQEQFNKLHPSYYYKANAAILVFDITRKITYKHLQDWFGELRQHVE-DVPAICVANKIDIDMTVTERNFAFPAR---------------------------HKL-PFYFVSASSGINVVKVFREALKLAVANEECPP-D-----------------------EVLDEIYRLLKSD-----D-----NKCA-------------------------------------------------------------------------------------------------------------------
VbrRABL2  -MSAPDTKSEPSGQ-------------------------LSTAQGDTLPEPDLKIILLGDSAVGKSKLVERFLLQNYNPRQLSTYALTLFRHYETI-------------DG----KRYSVDFWDTAGQEQFDHLHPSYYYRADACILAFDCTRKGTYKSLDKWYRELREYRP-DIPCILVANKIDVDESVTRKRFQFATN---------------------------NSL-PFYFVSASNGTNVVRVFREAIQLAVHHQLHPK-D-----------------------EVTSEILRLLRSD-----D-----HTAAPNAP-----------------PFDPPLSPSPS-HPSSRQSPHQPPPEG-----------GLMGQGHPRGLVSVSESSRAGQQW--------RENG--------------------
CveRABL2  -MTTSKTAAEEAEK-------------------------RRLALQADLPDADLKIILLGDSNVGKSKLVERFLLDDYNPRMLSTNALTMFRYNTKI-------------DS----KKVAVDFWDTAGQEDFDHLHPSYYFQANACILTFDVTRRDSYKSLPKWYQELRAFCP-DIPCLLVANKIDEDMKSTSKKWAFAET---------------------------HEM-PLFFVSAADGTNVVRVFREAIQLAHHFKENSK-D-----------------------GVMDELLTLLKEE-----K-----LDDLADLEV----VGNPSSS---------------------------------------------------------------------------------------------------
BnaRABL2  -MDAPESKSTSA-----------------KEDKPAEKSGNQGETTEGDGKSGIKVILLGDSGVGKSKLIERFLMDKYVPLQNSTYALTIFRHKAKV-------------GE----KEVDIDFYDTAGQERFASMHPSYYFRAQSCVLVFDTTRKITYKNLMQWYKELKQYRP-GIPIICVANKIDVNPKATTKKFKFATS---------------------------RDL-PLYYCSAADGTNVVRLFKEAIRLAVEGAEKPS-N-----------------------DFTEEVMRTLEYF-----D-----MKEKTQ---------VEGND------KDKDKEKSNTETIKEKPKN--------------------------------------------------------------------------
RfiRABL2  -MSK-----------------------------------DNNESSEQLEQADLKIILLGDSAVGKSKLMERFLLDNYEPKQLSTYALTIYRHTTTV-------------ND----KPLLVDFWDTAGQERFNSMHPSYYHRAHACILVFDCKRKQTYQNLNSWYNELLQYRG-VIPTIVIGNKIDCDVSVTKKKFQFAEK---------------------------RKL-PIDFTSAADGTNVVKVFNKIVDMAFQFKQNPT-D-----------------------NSFEQILQVLDLL-----P----------------------------------------------------------------------------------------------------------------------------
PbrRABL2  -MS------------------------------------AEKATADTTMEAELKIILLGDSAVGKTKLVERYLMDNYEPQQLSTYALTVFRHNTEI-------------DG----KPVSIDFWDTAGQERFTSMHPSYYYRAHACILAFDVTRKVTYQNMNAWYKELRQYRP-KIPVIVVANKIDSDYKVTEKTFAFATK---------------------------RKL-PFFFCSASDGTNVVTVFKEAIKAAMEFKSLPS-G-----------------------DFMDDVLETVAYF-----D-----AKSKGR---------HAA--------SASGKGEDND-DDIEITA---------------------------------------------------------------------------
```
